# Oral Ketamine vs Placebo in Patients With Cancer-Related Neuropathic Pain: A Randomized Clinical Trial

**DOI:** 10.1001/jamaoncol.2018.0131

**Published:** 2018-04-05

**Authors:** Marie T. Fallon, Andrew Wilcock, Caroline A. Kelly, James Paul, Liz-Anne Lewsley, John Norrie, Barry J. A. Laird

**Affiliations:** 1 Edinburgh Cancer Research Centre (IGMM), University of Edinburgh, Edinburgh, United Kingdom.; 2 Faculty of Medicine and Health Sciences, University of Nottingham, Nottingham, United Kingdom; 3 Cancer Research UK Clinical Trials Unit, Institute of Cancer Sciences, University of Glasgow, Glasgow, United Kingdom; 4 Usher Institute, University of Edinburgh, Edinburgh, United Kingdom

## Abstract

This multicenter randomized clinical trial compares oral ketamine with placebo for treating neuropathic pain in patients with cancer.

Ketamine hydrochloride is used as an adjuvant treatment for cancer-related neuropathic pain, but evidence of its effectiveness is limited.^1^ Findings of a large trial investigating the use of ketamine for general cancer pain were negative, but the population studied did not specifically have neuropathic pain.^2^ A randomized trial of oral ketamine for cancer-related neuropathic pain has been called for,^3^ and the present trial addresses that need.^1^

## Methods

A multicenter, double-blind randomized clinical trial of oral ketamine vs placebo was conducted in the United Kingdom cities of Edinburgh, Glasgow, Nottingham, and Lancashire in adults with cancer-related neuropathic pain, which was defined using set criteria (Leeds Assessment of Neuropathic Symptoms and Signs). Patients had previously been treated with adjuvant analgesics for neuropathic pain, which had been ineffective or suboptimal. Preexisting analgesia was continued throughout the trial. Patients were centrally randomized using minimization, then ketamine or placebo was titrated across 2 weeks to an effective and tolerable dosage (Figure). The starting dosage was 40 mg/d, with a maximum dosage of 400 mg/d. Patients continued to receive a stable dosage for 16 days. Patients who did not experience an analgesic benefit were withdrawn from the study. The study was approved by the West of Scotland Multicentre Research Ethics Committee (the full trial protocol is available in the Supplement; isrctn.org Identifier: ISRCTN49116945 and clinicaltrialsregister.eu Identifier: 2007-002080-27), and participants provided written informed consent.

**Figure.**
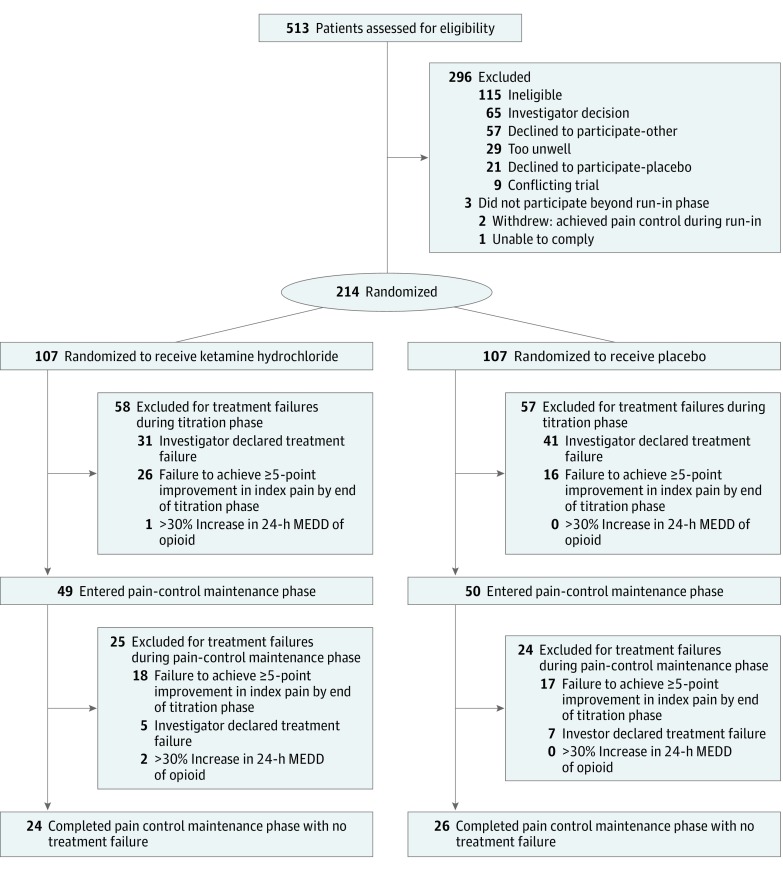
CONSORT Study Flow Diagram Among the 107 patients randomized to ketamine, the median (interquartile range [IQR]) time spent in the titration phase for the 58 patients who experienced treatment failure and were excluded was 14 (14-14) days; range, 7 to 28 days. Of the 49 patients who entered the pain-control maintenance phase, the median (IQR) time spent in the titration phase was 14 (14-15) days; range, 8 to 21 days. Among the 107 patients randomized to placebo, the median (IQR) time spent in the titration phase for the 57 patients who experienced treatment failure and were excluded was 14 (12.5-14.0) days; range, 0 to 22 days. Of the 50 patients who entered the pain-control maintenance phase, the median (IQR) time spent in the titration phase was 14 (14-15) days; range, 5 to 20 days. MEDD indicates morphine-equivalent daily dose.

The primary end point was duration of analgesic benefit, defined as an improvement of 5 points or more in the index pain score (using the Sensory Component of the Short Form McGill Pain Questionnaire), compared with the baseline score during the 16 days of receiving a stable dosage of ketamine or placebo. Patients in whom titration failed were considered to have a duration of 0 days. Maintenance of analgesic benefit was considered to have failed in patients whose opioid dosage increased during this time. Secondary end points included mean and worst pain; mood (Hospital Anxiety and Depression Score, a self-administered anxiety and depression screening tool for use in nonpsychiatric patients. The tool has 14 items, which focus on the emotional and cognitive aspects of each aspect. Each item is scored from 0 to 3 for a combined maximum of 21 for each aspect, with higher scores reflecting a higher symptom load); mean change in global distress in the last 24 hours (National Comprehensive Cancer Network Distress Thermometer, which uses a scale of 0 to 10, where 0 is no distress and 10 is extreme distress); quality of life (EuroQoL Thermometer, a patient-rated assessment of present quality of life comprising 5 questions, each assigned a value of 0 to 2 points, with higher values representing better quality of life. The sum of these responses produces a score on a scale of 0 to 10, which is then translated into a scale of 0 to 100); and serious adverse events (National Cancer Institute, Common Toxicity Criteria for Adverse Events version 3.0, which provides clinicians with descriptive terminology for reporting adverse events. Each adverse event is graded on a scale of 1 to 5, with higher grades representing greater severity).

An intention-to-treat approach was used, with a sample size of 107 patients per arm providing 80% or greater power to detect an improvement in the duration of analgesic benefit while receiving ketamine corresponding to a 20% increase in patients in whom analgesic benefit was maintained at 16 days (maximum hazard ratio [HR], 0.58). To compare duration of analgesic benefit, we used Cox proportional hazards regression with a confirmatory log-rank test. Secondary end points were analyzed using parametric and nonparametric methods. Quality of life data were analyzed by calculating the mean of the area under the curve over the time in the study after adjusting for the baseline value. All *P* values are 2-tailed, and *P* < .05 was statistically significant. Statistical analyses were performed using SPSS version 22.0 for Windows (IBM).

## Results

Two hundred fourteen patients (median [IQR] age, 58 [51-66] years; 141 [65.8%] female) were randomized, with comparable demographic features between arms (Figure). A variety of cancer types were represented; however, in 160 patients (74.7%) the cancer was in remission, and most of these patients had chronic, chemotherapy-induced, neuropathic pain. Two hundred nine patients (97.6%) were following treatment regimens for neuropathic pain. Data on duration of neuropathic pain were not collected. The median morphine-equivalent daily dose for both arms was 0 mg. There was no difference in the duration of analgesic benefit between arms as assessed by the adjusted (minimization factors) Cox proportional hazards model (ketamine to placebo HR, 0.95 (95% CI, 0.70-1.29); *P* = .75), supported by the log-rank test (*P* = .69). The median duration of analgesic benefit was 0 days (95% CI, 0-1 day) for ketamine and 0 days (95% CI, 0-4 days) for placebo. To illustrate, 34 of 107 patients (31.8%) receiving ketamine vs 39 of 107 (36.4%) receiving placebo maintained analgesic benefit at day 4 of the stable dosage (95% CI for difference, −17% to 8%). Corresponding figures at day 16 were 24 of 107 patients (22.4%) receiving ketamine and 27of 107 (25.2%) receiving placebo (95% CI for difference, −14% to 9%). There were no differences between arms among the secondary outcomes (Table).

**Table.** Secondary Outcomes and AUC Analyses for Distress, Quality of Life, and Mood

Variable	Ketamine Hydrochloride	Placebo	Median Difference, Ketamine − Placebo (95% CI)^a^	*P* Value		
				Secondary Outcomes	AUC Analyses	
					Unadjusted^b^	Adjusted^c^
**Secondary Outcomes**						
Patients with analgesic benefit at day 4, %	34 (31.8)	39 (36.4)	NA	.47	NA	NA
Patients with an analgesic benefit at day 16, %	24 (22.4)	27 (25.2)	NA	.63	NA	NA
**AUC Analyses**^d^^,^^e^						
NCCN Distress Thermometer	−2.972	−3.053	0.081 (−0.500 to 0.833)	NA	.64	.92
EuroQoL Thermometer	−17.756	−20.279	2.523 (−5.667 to 9.250)	NA	.89	.92
Hospital Anxiety and Depression Score						
Anxiety score	−3.605	−3.625	0.020 (−1.417 to 1.250)	NA	.65	.92
Depression score	−3.481	−3.654	0.173 (−0.500 to 0.958)	NA	.66	.92

Abbreviations: AUC, area under the curve; NA, not applicable; NCCN, National Comprehensive Cancer Network.

^a^ Estimated using 100 000 bootstrap samples.

^b^ Determined using the Mann-Whitney test.

^c^ Adjusted for multiple testing (false-discovery rate method).

^d^ Missing data were imputed (last observation carried forward) for the AUC analyses only.

^e^ Descriptions, scales, and possible range of scores for the NCCN Distress Thermometer, EuroQoL Thermometer, and Hospital Anxiety and Depression Score can be found in the Methods section of this research letter.

There were 18 serious adverse events: 8 in patients receiving ketamine and 10 in patients receiving placebo. Common adverse events were cognitive disturbance, dizziness, fatigue, nausea, and somnolence.

## Discussion

This trial reports that ketamine was equivalent to placebo for cancer-related neuropathic pain. Findings enhance previous work^4^ by examining ketamine in cancer-related neuropathic pain. There may be subgroups of patients for whom ketamine is helpful, such as those with central sensitization. A limitation of the present study was that we did not specifically select patients with clinical evidence of central sensitization, for whom it is reasonable to hypothesize a more specific analgesic target for ketamine. Future studies that examine ketamine in chronic neuropathic pain should focus on patients with central sensitization, which can be established by a bedside test. This approach would be congruent with preclinical knowledge and would address an important remaining unanswered question.^5^

## Notes

Supplement. Trial Protocol.
