# Supplementary material for: Oral Ketamine vs Placebo in Patients With Cancer-Related Neuropathic Pain: A Randomized Clinical Trial
Source: JAMA Oncol. 2018 Apr 5;4(6):870–2. doi: 10.1001/jamaoncol.2018.0131 (PMC6145686; doi:10.1001/jamaoncol.2018.0131)
Supplement: Supplement. — Trial Protocol. [file jamaoncol-4-870-s001.pdf]

K.P.S.

(**K**etamine in **P**ain **S**tudy)

# A randomised double-blind controlled trial of ketamine versus placebo in conjunction with best pain management in neuropathic pain in cancer patients

| | |
|---|---|
| Protocol number: | KPS 2008-01 |
| Date: | 13/03/2012 |
| Version: | 3.1 |
| EudraCT number: | 2007-002080-27 |
| ISCRTN number: | ISCRTN49116945 |

This study is jointly sponsored by The University of Glasgow and NHS Greater Glasgow and Clyde

# PROTOCOL APPROVAL SIGNATURE PAGE

**Chief Investigator:** **Professor Marie T Fallon**
Palliative Medicine/Oncology
School of Molecular and Veterinary Medicine
University of Edinburgh
Edinburgh Cancer Centre
Western General Hospital
Crewe Road
Edinburgh
EH4 2XU

**Signature:** ......................................................

**Date (dd/mon/yyyy)** ......................................................

**Participating Site**

**Site Name:** ......................................................

**Principal Investigator:** ......................................................

**Signature:** ......................................................

**Date (dd/mon/yyyy):** ......................................................

## Chief Investigator

**Professor Marie T Fallon**
Institute of Genetics and Molecular Medicine
Edinburgh Cancer Research Centre (CRUK)
Western General Hospital
Crewe Road
Edinburgh EH4 2XR

Tel: 0131 777 3518
Fax: 0131 777 3564
Email: marie.fallon@ed.ac.uk

## Trial Development and Co-Chief Investigator

**Dr Barry J A Laird**
Institute of Genetics and Molecular Medicine
Edinburgh Cancer Research Centre (CRUK)
Western General Hospital
Crewe Road
Edinburgh EH4 2XR

Tel: 0131 777 3548
Fax: 0131 777 3564
Email: barry.laird@ed.ac.uk

## Principal Investigators

**Dr Barry Laird**
Dept of Palliative Medicine
The Beatson West Of Scotland Cancer Centre
1053 Great Western Road
Glasgow G12 0YN

Tel: 0141 211 7406
Fax: 0141 201 3418
Email: barry.laird@ed.ac.uk

**Dr Alison Parr**
St Catherine's Hospice
Lostock Lane, Lostock Hall
PRESTON
PR5 5XU

Tel: 01772 629171
Fax: 01772696399
Email: Alison.parr@lthtr.nhs.uk

**Dr Patrick Stone**
St George's Healthcare NHS Trust
Blackshaw Road, Tooting
LONDON SW17 0QT

Tel: 02087253186
Fax: 02087252473
Email: patrick.stone@stgeorges.nhs.uk

**Dr Andrew Wilcock**
Nottingham University Hospitals
NHS Trust
Hucknall Rd, NOTTINGHAM, NG5 1PB

Tel: 0115 9627 778
Fax: 0115 9627 779
Email: Andrew.Wilcock@nottingham.ac.uk

## Trial Statistician

**Caroline Bray**
Senior Statistician
Cancer Research UK Clinical Trials Unit, Level 0
The Beatson West Of Scotland Cancer Centre
1053 Great Western Road
Glasgow G12 0YN

Tel: 0141 301 7198
Fax: 0141 301 7244
Email: caroline.bray@glasgow.ac.uk

**Project Manager**

**Liz-Anne Lewsley**
Project Manager
Cancer Research UK Clinical Trials Unit, Level 0
The Beatson West Of Scotland Cancer Centre
1053 Great Western Road
Glasgow G12 0YN

Tel: 0141 301 7193
Fax: 0141 301 7244
Email: Liz-Anne.lewsley@glasgow.ac.uk

**Pharmacovigilance/Quality Assurance**

**Lindsey Connery**
Pharmacovigilance/QA Manager
Cancer Research UK Clinical Trials Unit, Level 0
The Beatson West Of Scotland Cancer Centre
1053 Great Western Road
Glasgow G12 0YN

Tel: 0141 301 7209
Fax: 0141 301 7213
Email: Lindsey.connery@glasgow.ac.uk

**Clinical Trial Monitor**

**Eileen Smillie**
Clinical Trial Monitor
Cancer Research UK Clinical Trials Unit, Level 0
The Beatson West Of Scotland Cancer Centre
1053 Great Western Road
Glasgow G12 0YN

Tel: 0141 301 7948
Fax: 0141 301 7213
Email: Eileen.smillie@glasgow.ac.uk

**Trial Co-ordinator**

**Diann Taggart**
Clinical Trial Co-ordinator
Cancer Research UK Clinical Trials Unit, Level 0
The Beatson West Of Scotland Cancer Centre
1053 Great Western Road
Glasgow G12 0YN

Tel: 0141 301 7234
Fax: 0141 301 7228
Email: diann.taggart@glasgow.ac.uk

**Trial Sites**

The Beatson West Of Scotland Cancer Centre
1053 Great Western Road
Glasgow G12 0YN

Tel: 0141 301 7000

Edinburgh Cancer Centre
Western General Hospital
Crewe Road
Edinburgh EH4 2XU

Tel: 0131 537 1000

## Trial Sites (Cont)

St Catherine's Hospice
Lostock Lane, Lostock Hall
PRESTON
PR5 5XU

Royal Preston Hospital (Pharmacy for St Catherine's Hospice)
Sharoe Green Lane
Fulwood
PRESTON PR2 9HT

Tel: 01772 629171

St George's Healthcare NHS Trust
Blackshaw Road
Tooting
LONDON SW17 0QT

Tel: 02087253186

Hayward House
Nottingham University Hospitals NHS Trust
Hucknall Road
Nottingham NG5 1PB

Tel: 01159627619

## Trial Management

Cancer Research UK Clinical Trials Unit, Level 0
The Beatson West Of Scotland Cancer Centre
1053 Great Western Road
Glasgow G12 0YN

Tel: 0141 301 7193

## Study Co-Sponsors

The co-sponsors of this clinical trial are NHS Greater Glasgow and Clyde and the University of Glasgow

Contact:-

Dr Nathaniel Brittain
NHS Greater Glasgow and Clyde
West R&D Office
1st Floor, Tennent Institute
Western Infirmary
Glasgow G11 6NT

Tel: 0141 211 8544
Email: nathaniel.brittain@ggc.scot.nhs.uk

## Production and Distribution of IMP

Kay Pollock
Pharmacy Production Unit
Western Infirmary
GLASGOW G11 6NT

Tel: 0141 211 2754
Email: kay.pollock@ggc.scot.nhs.uk

## STUDY SUMMARY

| | |
|---|---|
| **STUDY TITLE:** | A randomised double-blind controlled trial of ketamine versus placebo in conjunction with best pain management in neuropathic pain in cancer patients. |
| **STUDY OBJECTIVES:** | **Primary Objective:**<br>To establish whether ketamine given in addition to best standard pain management improves malignant neuropathic pain compared to best standard pain management alone. This is assessed using the sensory component of the McGill Short Form Questionnaire (appendix 4).<br>**Secondary Objectives:**<br>• To compare initial treatment benefit (at day 4 of assessment period of 16 days) using the sensory component of the McGill Short Form Questionnaire.<br>• To compare difference in overall pain between the study arms based on the VAS score (appendix 3).<br>• To compare difference in neuropathic pain between the study arms based on the LANSS pain scale (appendix 2).<br>• To assess worst pain score (index neuropathic site) between the two arms.<br>• To compare patient distress between the two arms based on NCCN (National Comprehensive Cancer Network) Distress Thermometer (appendix 5).<br>• To assess the side-effects and tolerability of trial drug.<br>• To assess the effect of intervention on quality of life scores (based on Euroqol thermometer, appendix 7), anxiety and depression (based on HAD scale, appendix 6) and opioid requirements.<br>• To assess the effect of intervention on sensory changes (as assessed by Quantitative Sensory Testing).<br>• To assess the effect of intervention on Breakthrough Cancer Pain (BTcP) using the Breakthrough Pain Questionnaire (Appendix 9). |
| **STUDY POPULATION:** | Patients with neuropathic pain of malignant origin, or related to treatment for malignancy, at all stages of disease. The sample size required for the study is 214. |
| **STUDY TREATMENT:** | Following a run-in period where opioid analgesia dose will be optimised, ketamine or placebo will be administered orally four times a day. The dose will be increased as per the titration schedule and dose increments will cease when pain allows or before then if toxicity is unacceptable. The assessment period will consist of the dose level reached during the titration schedule and will last for 16 days. Following this, a 7 day run-out period will be completed. |

**CONTENTS**


**1 STUDY BACKGROUND ... 9**
1.1 Neuropathic Pain ... 9
1.2 NMDA Receptor Antagonists ... 9
1.3 Ketamine ... 9
1.4 Pilot Study ... 9
1.5 Rationale For Study ... 9
1.6 Previous Work ... 9
**2 TRIAL OBJECTIVES AND DESIGN ... 10**
2.1 Primary Objective ... 10
2.2 Secondary Objectives ... 10
2.3 Trial Design ... 10
*2.3.1 Stage 1 Run-in Period ... 12*
*2.3.2 Stage 2 Titration Period ... 12*
*2.3.3 Stage 3 Assessment Period ... 13*
*2.3.4 Stage 4 Run-out Period ... 13*
*2.3.5 Blood Sampling ... 13*
*2.3.6 Schedule of Pain Assessments ... 14*
**3 PATIENT SELECTION ... 15**
3.1 Patient Registration and Randomisation ... 15
3.2 Eligibility Criteria ... 15
*3.2.1 Inclusion Criteria ... 15*
*3.2.2 Exclusion Criteria ... 15*
3.3 Recruitment ... 15
3.4 Withdrawal Criteria ... 15
**4 TREATMENT ... 16**
4.1 Trial medication ... 16
*4.1.1 Route of Administration ... 16*
*4.1.2 Dosage Regimen ... 16*
*4.1.3 Side effects ... 17*
*4.1.4 Dose Reduction ... 17*
*4.1.5 Drug supply ... 17*
*4.1.6 Lifestyle Requirements ... 18*
4.2 Concomitant Medication ... 18
4.3 Treatment following study completion ... 18
4.4 Emergency Unblinding ... 18
**5 ASSESSMENT OF EFFICACY ... 19**
5.1 Efficacy Parameters ... 19
*5.1.1 Primary Efficacy Parameters ... 19*
*5.1.2 Secondary Efficacy Parameters ... 19*
*5.1.3 Procedures for Assessing Efficacy Parameters ... 19*
**6 SAFETY ASSESSMENT ... 20**
6.1 Definition of an Adverse Event ... 20
6.2 Adverse Event Reporting ... 20
6.3 Definition of a Serious Adverse Event ... 20
6.4 Definition of a Serious Adverse Reaction ... 21
6.5 Definition of Suspected Unexpected Serious Adverse Reactions (SUSARS) ... 21
6.6 Definition of NIMPs (Non Investigational Medicinal Products) ... 21
6.7 When SAEs are Not Required to be Reported ... 21
6.8 Procedure for reporting SAEs ... 22
6.9 Procedure for Identifying SUSARS ... 22
6.10 Procedure for Reporting SUSARS ... 22
6.11 Annual Safety Reports ... 23
**7 STATISTICS ... 23**
7.1 Sample Size ... 23
7.2 Analytical Plan ... 23
*7.2.1 Primary efficacy analysis ... 23*
*7.2.2 Secondary efficacy analysis ... 24*
*7.2.4 Safety Analysis ... 24*
*7.2.5 Interim Analysis ... 24*
**8 REGULATORY ISSUES ... 24**
8.1 Clinical Trials Authorisation (CTA) ... 24
8.2 Ethics Approval ... 24
8.3 Informed Consent ... 24
8.4 Patient Confidentiality ... 25
8.5 Liability, Indemnity and Insurance ... 25
8.6 Funding ... 25
8.7 Monitoring, Audits and Inspections ... 25

**9 TRIAL MANAGEMENT AND DATA COLLECTION ... 26**
9.1 Data Collection ... 26
9.2 Trial Management Group ... 26
9.3 Trial Steering Committee ... 26
9.4 Data Monitoring and Ethics Committee ... 26
**10 PUBLICATION AND AUTHORSHIP ... 26**
**11 QUALITY ASSURANCE ... 27**
**12 ALLOCATION OF STUDY RESPONSIBILITIES ... 27**
12.1 Co-Sponsor Responsibilities (NHS Greater Glasgow and Clyde/University of Glasgow) ... 27
12.2 Clinical Trials Unit (CTU) ... 28
12.3 Chief Investigator (CI) ... 28
12.4 Participating Site ... 28
12.5 Principal Investigator (PI) ... 28
**13 DEFINITION OF END OF STUDY ... 28**
**14 REFERENCES ... 28**
**APPENDIX 1: OPIOD TITRATION SCHEDULE ... 31**
**APPENDIX 2: THE LANSS PAIN SCALE ... 32**
**APPENDIX 3: VISUAL ANALOGUE SCORE (VAS) ... 34**
**APPENDIX 4: MCGILL SHORT FORM PAIN QUESTIONNAIRE ... 35**
**APPENDIX 5: NCCN DISTRESS THERMOMETER ... 36**
**APPENDIX 6: HOSPITAL ANXIETY AND DEPRESSION SCALE ... 37**
**APPENDIX 7: EUROQOL THERMOMETER ... 39**
**APPENDIX 8: QST ... 40**
**APPENDIX 9: BREAKTHROUGH PAIN QUESTIONNAIRE ... 41**
**APPENDIX 10: PHARMACOGENETIC BLOOD SAMPLING ... 42**
**APPENDIX 11: RUN OUT SCHEDULE ... 43**
**APPENDIX 12: DECLARATION OF HELSINKI 2000 ... 45**

# 1 STUDY BACKGROUND

## 1.1 Neuropathic Pain

Neuropathic cancer pain is a significant clinical challenge for which standard treatments are often suboptimal and it remains one of the most common symptoms in cancer sufferers. Studies suggest it occurs in up to 40% of such patients whilst at least 50% of all difficult to manage cancer pain, is neuropathic in origin[1, 2].

One of the main reasons neuropathic pain is a challenge is because of the unacceptable opioid side effects at doses required to control the pain. Commonly used adjuvant analgesics have a number needed to treat (NNT) of three which means two-thirds of patients will fail on any given adjuvant chosen. Furthermore adjuvant combinations cause an increased side effect profile. Uncontrolled neuropathic pain is associated with anxiety, depression and reduced quality of life.[3,4] It is a common cause of hospital and hospice admission and of referral for an anaesthetic intervention.

## 1.2 NMDA Receptor Antagonists

Animal studies in the late eighties provided early indications of the mechanisms of neuropathic pain and the need to treat this as a separate entity from other types of pain. N-methyl-D-aspartate (NMDA) receptors within the spinal cord have a documented role in the patho-physiology of neuropathic pain.

## 1.3 Ketamine

Subsequent human studies have established ketamine as a proven non-competitive antagonist of the NMDA receptor ion channel within the spinal cord. Ketamine blocks the NMDA receptor which subsequently acts by "winding down" and minimising pain transmission; which is particularly of benefit when a hyperexcitability state exists, commonly present in neuropathic pain states.

It has a proven role in neuropathic pain[5] and pain secondary to critical limb ischaemia[6], however its use in neuropathic pain of malignant origin remains unsubstantiated. Through case reports some clinicians who are expert in the use of ketamine have reported good pain relief in situations where best standard approaches have failed. Examination of the use of ketamine in an objective, systematic fashion should enable equity of access to this treatment, if shown to be effective in a randomised controlled trial.

## 1.4 Pilot Study

Recently we have completed a pilot study with s-ketamine, racemic ketamine and placebo in 65 patients. This double-blind randomised placebo controlled trial provides supporting evidence that ketamine may be superior to placebo in malignant neuropathic pain.[7]

## 1.5 Rationale For Study

Following on from our pilot work, a definitive double-blind randomised controlled trial can now be conducted comparing ketamine with placebo, against a background of best standard analgesic management.

## 1.6 Previous Work

Medline, Embase and Cochrane library have been searched and systematic reviews were located using racemic ketamine.

Three systematic reviews using racemic ketamine were identified. Bell et al completed a review of the evidence for ketamine as an adjuvant to opioids for cancer pain[8]. Quantitative meta-analysis was not possible due to insufficient numbers however the two included studies concluded that ketamine improves morphine treatment in cancer pain.

A further two systematic reviews were identified. Elia et al examined the role of ketamine in postoperative pain whilst Subramaniam et al examined the role of ketamine as an adjuvant analgesic to opioids [9,10]. These were supportive of the use of ketamine, however no trials were identified supporting the use of ketamine in neuropathic pain of malignant origin.

A review of the above has been published by the Chief Investigator.[11]

International Cancer Research Portfolio in cancer and the metaRegister of controlled trials (http://www.controlled-trials.com/mrct) have been searched and there are no current trials involving ketamine in cancer related neuropathic pain.

# 2 TRIAL OBJECTIVES AND DESIGN

## 2.1 Primary Objective

To establish whether ketamine given in addition to best standard pain management improves malignant neuropathic pain compared to best standard pain management alone. This is assessed using the sensory component of the McGill Short Form Questionnaire (appendix 4).

## 2.2 Secondary Objectives

- To compare initial treatment benefit (at day 4 of assessment period of 16 days) using the sensory component of the McGill Short Form Questionnaire[12] (appendix 4).
- To compare difference in overall pain between the study arms based on the pain intensity visual analogue score (VAS score [13,14]) (appendix 3).
- To compare difference in neuropathic pain between the study arms based on the Leeds Assessment of Neuropathic Symptoms and Signs (LANSS [15,16,17,18]) pain scale (appendix 2).
- To assess worst pain score (index neuropathic site) between the two arms.
- To compare patient distress between the two arms based on NCCN Distress Thermometer [19,20] (appendix 5).
- To assess the side-effects and tolerability of trial drug.
- To assess the effect of intervention on quality of life scores (based on Euroqol thermometer[21], appendix 7), anxiety and depression (based on HADS[22], appendix 6) and opioid requirements.
- To assess the effect of intervention on sensory changes (as assessed by Quantitative Sensory Testing (QST) Appendix 8).
- To assess the effect of intervention on Breakthrough Cancer Pain (BTcP) using the Breakthrough Pain Questionnaire (Appendix 9).

## 2.3 Trial Design

The trial will be of a randomised double blind design and incorporate a placebo control arm. This control is both essential and acceptable for this type of study. The pilot study showed that patients in the placebo arm showed clinical signs of improvement thus it can be expected that patients randomised to the placebo arm are likely to have a clinical benefit from being in the study. Nevertheless it should be emphasized that both arms will receive best supportive care throughout the entire duration of the study.

The trial design also incorporates a "run-in" period. This is a vital component of the design. It allows for standardization of the treatment groups prior to treatment intervention and for breakthrough analgesia during the study period. This has been used in previous studies.[6]

107 patients will be recruited to each treatment arm.

The trial will be carried out in 4 stages (see chart 1 on next page):

## *Chart 1 – Study Stages*

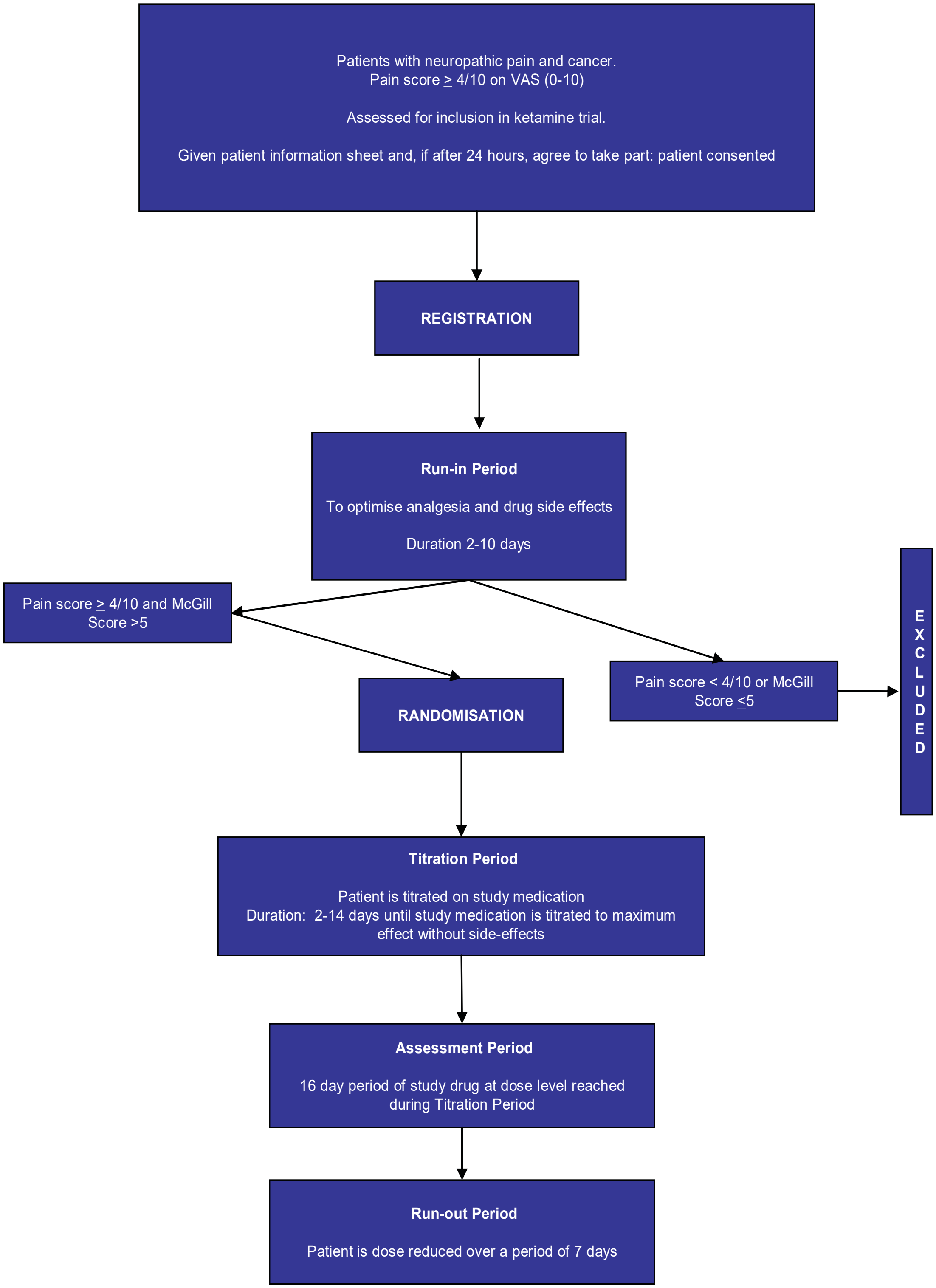

### *2.3.1 Stage 1 Run-in Period (Variable up to 10 days)*

Suitable patients will be identified and informed consent sought. It is expected that the vast majority of patients will already be taking adjuvant analgesics for neuropathic pain and doses of adjuvant drugs will not be altered or new medication added.

Anticonvulsants and antidepressants commonly used in the management of neuropathic pain rarely have a standard dose and indeed are tailored to the individual. Therefore it would be impossible to standardise an optimisation regimen for adjuvants as these are very much dependent on the individual. All patients who are entered into the study will have had or been offered a trial of, or currently be taking appropriate adjuvant analgesics (amitriptyline or gabapentin/pregabalin) and by definition will have failed on these or have sub-optimal pain scores, with worst pain (index site) remaining at $\geq$ 4/10 on a VAS. If the patient has not had a trial of either of these drugs, then this will have to have been patient choice.

During the run-in period the opioid dose will be optimised under a defined schedule (see appendix 1). This is essential as optimisation of opioid dose is fundamental to a valid analysis. This "run-in" period is likely to vary but expected to be a maximum of 10 days. During this time opioid dose adjustment will be done under the guidance of a research nurse and other investigators.

The ultimate aim of this run-in period is to ensure that both groups are on an optimised and stable regimen prior to intervention (stable regimen defined as the same dose of controlled release and no more variation than 2 breakthrough opioid doses over the normal for that patient for a period of 48 hours: two days without a change in opioid dose is sufficient to consider the opioid dose to be stable). If there is evidence of opioid toxicity, the opioid dose will be reduced as per standard clinical practice.

The variable run-in period is justified in that opioid requirements need to be optimised in order to have the best chance of determining the ketamine effect; if a high proportion of patients had to have their opioid dose increased between end of run in and assessment period then the ketamine effect would be very difficult to disentangle. In the analysis, patients who have their opioid dose modified are counted as "failures"; the aim is to ensure that patients are not counted as failures simply because they weren't getting the correct opioid dose in the first place. While this optimisation will usually be possible over a few days, up to 10 days for this "run-in" period is allowed.

Such opioid optimisation may result in significant improvement in pain, thus reassessment will take place at end of the run-in period and if scores are deemed to have improved (worst pain (index site)) less than 4/10 on a VAS in the past 24 hours or $\leq$5 McGill Sensory Scale Score) then such patients are no longer eligible and will be withdrawn from the study.

Although it is envisaged that the majority of patients will be taking opioid analgesia, this is not mandatory. Any patients who are not taking opioid analgesia will be due to patient preference, side-effects or a combination of these reasons.

### *2.3.2 Stage 2 Titration Period (Variable up to 14 days)*

Patients will then be randomised and enter the study period. Once a patient enters the titration period no new analgesic medications will be added during the titration phase, but alteration or addition of other medications which do not have any analgesic qualities will be permitted. Patients will be allowed breakthrough opioids at any point. If clinical need dictates, then analgesic doses will be increased or other analgesics added but this will count as a treatment failure.

Patients will then be titrated on either ketamine or placebo. This will be standardised as per a titration schedule (see section 4.1.2) with identical titration of placebo. Titration stops at a level when analgesia is achieved or individual side effects appear. Analgesia will be classified as having been achieved when there is a drop of 5 points or more in the McGill Sensory Pain Score (SF-MPQ) AND it is the opinion of the investigator that clinically meaningful analgesia has been attained. Titration will be performed under close supervision under the guidance of the Research Team. Patients will contacted daily by telephone during this period.

Patients must remain on any one dose level for 48 hours during the titration period to allow time for effect (patients can stay on any one dose level for longer than 48 hours at the discretion of the Investigator). If necessary the dose level can be decreased to the previous level at any juncture.

The titration period is variable and this is essential. In this situation ketamine should be considered as an analgesic like paracetamol or morphine in that it will either give or not give pain relief as seen in a drop in the pain scores (VAS and McGill). A long period to assess such pain relief is not needed, however to properly assess any improvement in pain relief due to ketamine the appropriate dose of ketamine for each patient must be reached: this is similar to the titration required for any other analgesic in cancer pain. This will be achieved during the titration period.

**Throughout the entire study, patients in both arms will be allowed breakthrough opioid medication, which will be recorded.**

#### *2.3.3 Stage 3 Assessment Period (Fixed – 16 days)*
Immediately after the ketamine/placebo titration period, pain assessment will be made.

The assessment period is four, 4-day periods of regular administration of the optimum dose of the trial medication reached during the preceding titration period.

Patients may decrease dose level (by no more than one dose level at any one time) during this period.

Where an assessment day (1, 4, 8, 12 or 16) falls on a weekend or holiday, the patient will be assessed the day prior or later (whichever is closest). For example, if assessment day falls on a Sunday, the patient will be seen on the Monday.

**During the whole study equal, identical regular assessment of both groups will be made continuing until the end of the study. Blinding will ensure that this occurs and will reduce the possibility of bias.**

Throughout the Titration and Assessment periods of the study, patients will be asked to complete a Patient Diary to record study drug taken.

#### *2.3.4 Stage 4 Run-out Period (Fixed – 7 days)*
Once the assessment period has been completed there will be a 7 day run-out period where trial medication will be titrated down.

#### *2.3.5 Blood Sampling*
Clinical work has highlighted that the way an individual responds to a medication is a complex process that has a genetic component. To this end in the future we intend to analyse the pharmacogenomics involved in pain states.

Trial participants will be asked to provide two (10ml) blood samples, one within the trial run-in period and a further sample during the assessment period. This is entirely optional and samples will be used for the assessment of pharmacogenomics at a later date (see Appendix 10).

### *2.3.6 Schedule of Pain Assessments*

| | Day | VAS | SF-MPQ | Toxicity | Opioid | HADS | QoL | Distress | LANSS | QST | BTPQ |
|---|---|---|---|---|---|---|---|---|---|---|---|
| **RUN IN** | 0 | X | X | | X | | | | X | | |
| | 1 | X | | | | | | | | | |
| | 2 | X | | | | | | | | | |
| | 3 | X | | | | | | | | | |
| | 4 | X | | | | | | | | | |
| | 5 | X | | | | | | | | | |
| | 6 | X | | | | | | | | | |
| | 7 | X | | | | | | | | | |
| | 8 | X | | | | | | | | | |
| | 9 | X | X | | X | X | X | X | X | X | X |
| **TITRATION** | 1 | X | X | X | X | | | | | | |
| | 2 | X | X | X | X | | | | | | |
| | 3 | X | X | X | X | | | | | | |
| | 4 | X | X | X | X | | | | | | |
| | 5 | X | X | X | X | | | | | | |
| | 6 | X | X | X | X | | | | | | |
| | 7 | X | X | X | X | | | | | | |
| | 8 | X | X | X | X | | | | | | |
| | 9 | X | X | X | X | | | | | | |
| | 10 | X | X | X | X | | | | | | |
| | 11 | X | X | X | X | | | | | | |
| | 12 | X | X | X | X | | | | | | |
| | 13 | X | X | X | X | | | | | | |
| | 14 | X | X | X | X | | | | | | |
| **ASSESSMENT** | 1 | X | X | X | X | X | X | X | X | | |
| | 2 | X | | X | X | | | | | | |
| | 3 | X | | X | X | | | | | | |
| | 4 | X | X | X | X | X | X | X | X | | |
| | 5 | X | | X | X | | | | | | |
| | 6 | X | | X | X | | | | | | |
| | 7 | X | | X | X | | | | | | |
| | 8 | X | X | X | X | X | X | X | X | | |
| | 9 | X | | X | X | | | | | | |
| | 10 | X | | X | X | | | | | | |
| | 11 | X | | X | X | | | | | | |
| | 12 | X | X | X | X | X | X | X | X | | |
| | 13 | X | | X | X | | | | | | |
| | 14 | X | | X | X | | | | | | |
| | 15 | X | | X | X | | | | | | |
| | 16 | X | X | X | X | X | X | X | X | X | X |

# 3 PATIENT SELECTION

## 3.1 Patient Registration and Randomisation

Patients must be registered with CRUK Clinical Trials Unit, Glasgow (telephone: 0141 301 7236/ fax: 0141 301 7228) prior to entry to the "run-in" period. Patient eligibility criteria will be checked and, if eligible, a 3-digit Screening Number will be allocated at this point.

The Trials Unit should be subsequently contacted to randomise patients who successfully complete the "run-in" period, have a stable opioid regimen and continue to fulfil the pain score and other study eligibility criteria. At this timepoint randomised patients will be allocated a 4-digit Trial Number. For patients who are not proceeding to randomisation, the reason for this will be recorded at this stage and no further data collected.

The patient randomisation will use minimisation with the following factors: centre, age, baseline McGill sensory pain score, gender and type of previous adjuvant analgesic for neuropathic pain. The minimisation algorithm will incorporate a random component.

## 3.2 Eligibility Criteria

### *3.2.1 Inclusion Criteria*

(1) ≥18 years of age.
(2) Patient has a histological proven cancer diagnosis.
(3) Written informed consent (to be obtained within 28 days prior to study entry).
(4) Index neuropathic pain (as defined by LANSS [15,16,17,18]) that is related to underlying malignancy or resulting from treatment received for this.
(5) Index neuropathic pain (worst pain) ≥ 4 on 0-10 (VAS).
(6) McGill Sensory Scale Score > 5.
(7) Patient has had a trial of at least one adjuvant analgesic (gabapentin, pregabalin, amitriptyline) or has been offered these and declined
(8) Patient is able to comply with study procedures.

### *3.2.2 Exclusion Criteria*

(1) Patients who have received chemotherapy or radiotherapy in the preceding six weeks that is likely to affect neuropathic pain.
(2) Patients who may have a change in tumoricidal treatment during the period of the study that is likely to alter pain during the course of the study.
(3) Diastolic blood pressure >100mmHg at screening (within 7 days).
(4) History of seizures in last 2 years.
(5) Patient is currently taking class I anti-arrhythmic drugs.
(6) Life expectancy less than two months.
(7) Patients who are actively hallucinating (visual or auditory within the last 48 hours).
(8) Women of childbearing potential not using adequate contraception.
(9) Patients with cerebrovascular disease (strokes).
(10) Patients with psychotic disorders or cognitive impairment.

## 3.3 Recruitment

The trial will run in 3 centres over 30 months. This equates to a required recruitment rate of 72 patients per centre or approximately 28 patients per year, per centre. This will be the only neuropathic pain study open to recruitment in each of these centres for the duration of the study recruitment period.

In each centre, the Hospital Palliative Care Team (HPCT) sees approximately 1200 patients per year, of which 500 would have neuropathic pain. As this trial is being overseen and recruitment is being pro-actively managed by the HPCT, it is expected that approximately 500 patients per centre per year will be screened. It is anticipated that 100 per year will be eligible. Allowing for a dropout rate of 10% during the run-in period (10% dropout was the rate in the pilot study) an accrual rate of 28 patients per centre per year is a realistic recruitment rate.

## 3.4 Withdrawal Criteria

The patient can decide to withdraw from the study at any time. The Principal Investigator (PI) also has the right to withdraw patients from the study if he/she feels that it is in the best interests of the patient. Full details of the reasons for withdrawal should be recorded

on the Case Report Form (CRF). Withdrawn patients should be followed-up in accordance with the protocol.

# 4 TREATMENT

## 4.1 Trial medication

**Ketamine Hydrochloride**

Molecular formula: $C_{13}H_{16}$CINO,HCl Molecular weight =274.2

CAS: 1867-86-9 (ketamine hydrochloride)

**Placebo**

This will be identical to the IMP in appearance and will not contain active substance.

### *4.1.1 Route of Administration*

Racemic ketamine commonly exists as a parenteral medication. However, there is a body of evidence which supports the use of ketamine injection used orally.[23,24,25]

Ketamine hydrochloride will be manufactured into immediate release capsules.

Ketamine Hydrochloride 1.15mg = 1mg ketamine base. Doses expressed in Table 1 (Dose Schedule) are the equivalent amount of ketamine base.

Identical placebo capsules will be provided marked with the same range of dose levels.

### *4.1.2 Dosage Regimen*

Study drug will be administered under a set-dosing regimen. Each dose will be administered four times daily between the hours of 08:00 and 22:00. First dose of study drug should be taken at night before bed. Under the dosing regimen study drug will be administered at seven dosing levels as shown below. Titration will stop when the McGill Pain Score drops by 5 points or side effects preclude further titration AND it is the opinion of the investigator that clinically meaningful analgesia has been attained. The dose level will be maintained for at least 48 hours before a further increment is made (patients can stay on any one dose level for longer than 48 hours at the discretion of the Investigator). It is envisaged that the majority of patients will reach dose level 4 and continue at this level for the duration of the study.

**Table 1: Dose Schedule**

| Day | Dose Level | Total Daily Dose (mg) |
|---|---|---|
| 1 | 1 | 40 |
| 2 | 1 | 40 |
| 3 | 2 | 80 |
| 4 | 2 | 80 |
| 5 | 3 | 120 |
| 6 | 3 | 120 |
| 7 | 4 | 160 |
| 8 | 4 | 160 |
| 9 | 5 | 240 |
| 10 | 5 | 240 |
| 11 | 6 | 320 |
| 12 | 6 | 320 |
| 13 | 7 | 400 |
| 14 | 7 | 400 |

If patients complete the trial they will remain on either ketamine or placebo for a period of between 17 days (minimum) and 30 days (maximum).

#### *4.1.3 Side effects*

Whilst ketamine in anaesthetic doses can cause extraneous muscle movements, cardiovascular stimulation and raised arterial pressure these are not usually problematic at proposed study doses. The main disadvantage of ketamine is the high incidence of hallucinations, nightmares, and other transient psychotic effects; these can be reduced when drugs such as haloperidol are also used. If necessary a supply of haloperidol will be issued at study site (Please refer to Protocol Section 6.6 – Non Investigations Medicinal Products).

Ketamine is **contra-indicated** in patients with uncontrolled hypertension and is best avoided in those prone to hallucinations or nightmares. It also has abuse potential and may itself cause dependence.

No side effects are expected from the placebo preparation.

#### *4.1.4 Dose Reduction*

If the patient has any adverse effects which are clearly attributable to the study drug, do not titrate further. If the patient finds adverse effects are intolerable, then reduce drug to previous Level (if on Level 1 then patient should STOP drug and withdraw from study).

During the Assessment Period patients can be dose reduced no more than one dose level at any time. For example, a patient on Dose Level 4 requiring reduction can only be reduced to Dose Level 3 not Dose Level 2. There is no limit to the amount of dose reductions a patient can have during the assessment period.

There will also be a run-out period whereby, depending on the dose level reached, the drug will be titrated **down** over 7 days (see **Table 2: Run Out Schedule Appendix 11)**. Patients who are withdrawn prior to completing the Assessment Period will be advised to titrate down their study drug with the guidance of the investigator.

#### *4.1.5 Drug supply*

**<u>Ketamine</u>**

For the purposes of the study, ketamine will be supplied as follows

Generic Name: Ketamine Hydrochloride

Pack: 10mg, 20mg, 40mg capsules

Manufacturer: Pharmacy Production Unit, Western Infirmary, Glasgow

Ketamine capsules will be administered orally. This will be supplied in an oral preparation which is appropriate and safe to be administered orally.

This will be bulked using lactose.

**<u>Placebo</u>**

This will be supplied by Fagron Pharmaceuticals and packaged /dispensed by the Pharmacy Production Unit, Western Infirmary in Glasgow. The placebo will be identical in appearance to the active drug and will contain lactose.

Study drug should be stored at room temperature (below $25^{0}$ C).

<u>Full drug accountability must be maintained throughout the study.</u>

**Study drug will be dispensed at study sites, the Pharmacy Production Unit will package placebo and active drug only.**

**Study drug will be marked for use in clinical trial patients only.**

#### *4.1.6 Lifestyle Requirements*
No restrictions will be placed on trial participants

### 4.2 Concomitant Medication
Patients will continue in their pre study medication during the course of the study period. Breakthrough doses of opioid analgesia will be permitted, however any alteration or introduction of other medication that may act either directly or indirectly to affect analgesia will not be allowed. If such an event occurs these patients will be withdrawn from the study.

Patients should not undergo chemotherapy or radiotherapy during the study period.

### 4.3 Treatment following study completion
Following study completion (completion of the assessment period, classification as “failures” or withdrawal) patients will be assessed for commencing ketamine off study. In patients who have a clinical benefit from the study drug, ketamine will be prescribed in the commercially available liquid form.

### 4.4 Emergency Unblinding
Emergency unblinding may take place in situations where the safe management of the patient’s medical condition **necessitates knowledge of the study medication by the person(s) responsible for the patients care**.

The trial allocation will only be revealed to individuals on a “need to know” basis and should never be revealed to the Study Statistician (apart from after the final study analysis).

In general, if time allows, the reason for emergency unblinding should be discussed initially with the Chief Investigator. However, if the case is a serious emergency where delay would put the patient’s well being at risk, this is not required.

Each site is required to provide a 24 hour emergency contact for unblinding.

Unblinding will be performed on request by the CTU IT Department via the Clinical Trial Co-ordinator (CTC). This service is available during office hours (0830 – 1700 Mon – Thurs and 0830 – 1630 Fri).

Sealed envelopes containing the code break for each individual bottle number will be supplied to site with each shipment of study drug. These should be held in the pharmacy site file and used as 24 hour emergency unblinding service (e.g. on-call pharmacist). **This method is <u>only to be used when unblinding via the CTU is not possible</u>**.

If a patient requires to be unblinded then this has to be documented with a signature from the following site staff (these do not necessarily have to be signed at the same time, this is to ensure that both are aware that the patient has been unblinded):

- Principal Investigator (PI)
- Pharmacist

A reason must be given on the unblinding log for the code break and the CTC at the CTU must be informed **<u>immediately</u>** that this has been performed. For unblinding that has been performed outside office hours then the CTC must be informed the next working day.

Each site will have an Unblinding Log which requires to be completed in the event of unblinding being performed. This is kept in the Investigator Site File.

All of this information will be captured on the Case Report Forms.

# 5 ASSESSMENT OF EFFICACY

## 5.1 Efficacy Parameters

### *5.1.1 Primary Efficacy Parameters*

The primary study end-point is time to treatment "failure". A treatment "failure" is defined as failure to achieve a 5-point drop in the sensory component of the SF-MPQ (Short-Form McGill Pain Questionnaire, appendix 4) from the end of the run in period (prior to randomisation) to any one of the assessment time points end of titration period, day 4, day 8, day 12, day 16). Patients who have had significant changes in opioid dose defined (greater than 30% increase in 24 hour mean equivalent daily dose [MEDD] during titration or assessment period) will also be counted as treatment "failures". All patients who withdraw from the study during the titration or assessment period for any reason (e.g. lack of efficacy, side effects) will also be counted as treatment "failures". Conversely a treatment "success" is a patient who remains on study with no significant change in opioid dose and has a 5-point drop in the sensory component of the SF-MPQ.

### *5.1.2 Secondary Efficacy Parameters*

- The initial treatment benefit (at day 4 of assessment period of 16 days) using the sensory component of the McGill Short Form Questionnaire.
- The difference in overall pain between the study arms based on the VAS score (appendix 3).
- The difference in neuropathic pain between the study arms based on the Leeds Assessment of Neuropathic Symptoms and Signs (LANSS) pain scale (appendix 2).
- The worst pain score (index neuropathic site) in the previous 24 hours (between the two arms) at study baseline and then during study assessment period
- Patient distress between the two arms based on NCCN Distress Thermometer (appendix 5).
- Side-effects and tolerability of trial drug.
- The effect of the intervention on quality of life scores (based on Euroqol thermometer, appendix 7), anxiety and depression (based on HAD scale, appendix 6) and opioid requirements.
- To assess the effect of intervention on sensory changes (as assessed by Quantitative Sensory Testing (QST) Appendix 8).
- To assess the effect of intervention on Breakthrough Cancer Pain (BTcP) using the Breakthrough Pain Questionnaire (Appendix 9)

### *5.1.3 Procedures for Assessing Efficacy Parameters*

The above tools are applied at the following time-points:-

1. SF-MPQ (sensory component) Start of run in period, end of run in period (prior to randomisation), daily throughout titration and days 1, 4, 8, 12 and 16 of assessment period.

2. VAS pain score – Daily throughout run in, titration and assessment period.

3. LANSS – Start of run in period, end of run in period (prior to randomisation), and day 1, 4, 8, 12 and 16 of assessment period.

4. Opioid use (24 hour oral morphine equianalgesic equivalent) – Start of run-in period, end of run-in period (prior to randomisation) and daily during titration and assessment periods.

5. HADS – anxiety/depression - End of run in period (prior to randomisation), and day 1, 4, 8, 12 and 16 of assessment period.

6. Euroqol thermometer – Quality of Life – End of run in period (prior to randomisation) and day 1, 4, 8, 12 and 16 of assessment period.

7. Distress – using NCCN Distress Thermometer End of run in period (prior to randomisation) and day 1, 4, 8, 12 and 16 of assessment period.

8. Breakthrough Cancer Pain – using the BTPQ End of run in period and day 16 of assessment period.

9. Sensory testing – using QST End of run in period and day 16 of assessment period.

## 6 SAFETY ASSESSMENT

Safety assessments will be performed in line with the guidance specified in The Medicines for Human Use (Clinical Trials) Regulations 2004.

### 6.1 Definition of an Adverse Event

An adverse event is any untoward medical occurrence in a subject to whom a medicinal product has been administered, including occurrences which are not necessarily caused by or related to that product.

### 6.2 Adverse Event Reporting

Adverse events will be recorded as they are reported, whether spontaneously volunteered or in response to questioning about well being at trial visits. The questioning about adverse events will cover the current visit as well as the period of time between the previous and the current visit. All adverse events will be documented in the subject's medical records and Case Report Form (CRF).

All adverse events must be followed until resolution, or for at least 30 days after discontinuation of study medication, whichever comes first or until toxicity has resolved to baseline or ≤ Grade 1, or until the toxicity is considered to be irreversible. Perceived lack of efficacy is not an adverse event.

An exacerbation of a pre-existing condition is an adverse event.

All adverse events and toxicities must be graded according to the NCI Common Toxicity Criteria for adverse events (NCI-CTCAE) Version 3.0 (http://ctep.cancer.gov/reporting/ctc.html).

Abnormal laboratory test results that are deemed clinically significant by the investigator and that lead to a change in the dosage of trial treatment or temporary or permanent discontinuation of trial treatment, or require intervention or diagnostic evaluation to assess the risk to the patient should be recorded as adverse events in the CRF and instigate further investigation and follow up as appropriate.

### 6.3 Definition of a Serious Adverse Event

A serious adverse event (SAE) is defined as any of the following, whether or not considered related to the trial treatment (trial treatment is ketamine hydrochloride or Placebo).

- Results in Death
- Life-threatening (i.e. at the time of the event)
- Requires inpatient hospitalisation or prolongation of existing hospitalisation
- Results in persistent or significant disability or incapacity
- Is a congenital anomaly/birth defect
- Is considered medically significant by the Investigator

Life threatening means that the patient was at immediate risk of death from the event as it occurred. It does not include an event that, had it occurred in a more serious form, might have caused death.

Requires in-patient hospitalisation should be defined as a hospital admission required for treatment of an adverse event.

Considered medically significant by the Investigator are events that may not result in death, are not life threatening, or do not require hospitalisation, but may be considered a serious adverse experience when, based upon appropriate medical judgement, the event may jeopardise the patient and may require medical or surgical intervention to prevent one of the outcomes listed above.

### 6.4 Definition of a Serious Adverse Reaction

A serious adverse reaction (SAR) is an SAE that may be related to trial treatment. The assessment of "relatedness" is primarily the responsibility of the Principal Investigator at site or agreed designee. SAEs that will be considered related will include any SAE that is documented as possibly, probably or definitely related to protocol treatment. The assessment of relatedness is made using the following:

| **Relationship** | **Description** |
|---|---|
| Unrelated | There is no evidence of any causal relationship. |
| Possible | There is some evidence to suggest a causal relationship (e, g. the event occurs within a reasonable time after administration of the trial medication). However the influence of other factors may have contributed to the event (e.g. the patient's clinical condition, other concomitant treatments). |
| Probable | There is evidence to suggest a causal relationship and the influence of other factors in unlikely. |
| Definitely | There is clear evidence to suggest a causal relationship and other possible contributing factors can be ruled out. |

### 6.5 Definition of Suspected Unexpected Serious Adverse Reactions (SUSARS)

A SUSAR is any serious adverse reaction that is unexpected. Unexpected is any reaction that is not a known reaction listed in the IMPD/Summary of Product Characteristics (SmPC).

### 6.6 Definition of NIMPs (Non Investigational Medicinal Products)

NIMPs are "Products which are not IMPs" and are referred to in Art. 2(d) of Directive 2001/20/EC and may be supplied to patients participating in a trial and used in accordance with the protocol. For instance, some clinical trial protocols require the use of medicinal products such as concomitant or rescue/escape medication for preventive, diagnostic or therapeutic reasons and/or ensure that ensure adequate medical care is provided for the subject. They may also be used in accordance with the protocol to induce a physiological response. These medicinal products do not fall within the definition of investigational medicinal products in Directive 200/20/EC and can be referred to as "non-investigational medicinal products" (NIMPs)."

Any SAE that could be the result of administration of a NIMP must be reported as an SAE. This can be an SAE related to the NIMP or a reaction between an IMP and NIMP. The NIMPs identified for the trial are **haloperidol** and **opioid analgesics**.

### 6.7 When SAEs are Not Required to be Reported

The following is a list of serious adverse events, which do not require to be reported as SAEs. These are:-

- Events based on the knowledge of the disease in question and expected.
- If a patient is admitted to hospital with a documented cancer related problem then this will not be reported as a SAE.

| **Events** | **Description** |
|---|---|
| Disease Related Events | • Hospitalisation or death due to disease progression<br>• Hospitalisation for planned investigations<br>• Hospitalisation for planned blood/platelet transfusion<br>• Hospitalisation for complications due to treatment, e.g. neutropenic sepsis |
| Other | • Hospitalisation for study drug administration, palliative care, terminal care or elective surgery |

### 6.8 Procedure for reporting SAEs

If a serious adverse event occurs that requires reporting, a Serious Adverse Event reporting form should be completed and faxed within 24 hours of becoming aware of the event to:

***Pharmacovigilance***
***Cancer Research UK Clinical Trials Unit, Level 0***
***The Beatson West Of Scotland Cancer Centre***
***1053 Great Western Road***
***Glasgow G12 0YN***

***Tel no. 0141 301 7209/7211***
***Fax no: 0141 301 7213***

**SAEs require to be reported once a patient has been registered and has started opioid titration *only for those events thought to be related to opioid analgesics.* Thereafter, once the patient has been randomised all SAEs should be reported as detailed below.**

The Chief Investigator will receive notification of all SAEs shortly after they are received by Pharmacovigilance and confirm agreement with the causality assessment made by the reporting Investigator.

A follow-up report should be completed when the SAE resolves or when additional information becomes available, unless the SAE is a suspected SUSAR when follow up information will be provided as requested by the CRUK Clinical Trials Unit and Chief Investigator.

SAEs are required to be reported for up to 30 days after discontinuation of study medication. Any SAE that occurs after 30 days post treatment (with no time limit) is also required to be reported if Investigators think the SAE is related to protocol treatment (is a SAR). Post treatment SARs should be reported by phoning the Cancer Research UK Clinical Trials Unit at the contact numbers noted above.

### 6.9 Procedure for Identifying SUSARS

The CRUK Clinical Trials Unit will prepare a SUSAR checklist for identifying potential SUSARS. The checklist is a list of the known expected reactions to ketamine hydrochloride against which a SAR can be checked. For any SARs not listed on the checklist the Chief Investigator will be contacted for an opinion of SUSAR status. The Chief Investigator is responsible for deciding if a SAR is a SUSAR.

Below is a list of known expected reactions to ketamine or analgesia:

| Expected Reactions | Description |
|---|---|
| Events due to ketamine or analgesia | Hospitalisation due to:<br>• Hypertension<br>• Tachycardia<br>• Psychotomimetic phenomenom (delirium, vivid dreams, nightmares, hallucinations, altered body image)<br>• Diplopia<br>• Nystagmus<br>• Clinical signs of opioid toxicity (myoclonic jerks, pseudo- hallucinations, hallucinations, vivid dreams) |

### 6.10 Procedure for Reporting SUSARS

The CRUK Clinical Trials Unit is responsible for the expedited reporting of all SUSARS to the MHRA, lead ethics committee, Principal Investigators at trial sites and the trial sponsor:

- Fatal or life threatening SUSARS will be reported within 7 days of the Trials Unit receiving the initial report. Any additional information will be reported within eight days of sending the first report.

- All other SUSARS will be reported within 15 days of the Trials Unit receiving the initial report.

### 6.11 Annual Safety Reports
Annual safety reports will be prepared and submitted by the CRUK Clinical Trials Unit Glasgow for all SARs reported for the trial. Annual Safety Reports will be submitted to the MHRA, lead ethics committee, and trial sponsor on the anniversary of obtaining the Clinical Trial Authorisation.

## 7 STATISTICS

### 7.1 Sample Size
The proposed sample size is 107 patients per arm. This sample size will provide at least 80% power to detect an increase in the "success" (see section 5.1.1) rate at day 16 of 20% on Ketamine compared to placebo over a range of possible placebo "success rates". These figures are based on the logrank test and a 5% two-sided level of statistical significance.

| Success rate at day 16 of assessment period | | Study Power |
|---|---|---|
| Placebo | Ketamine | |
| 10% | 25% | 90% |
| 10% | 30% | 98% |
| 15% | 30% | 82% |
| 15% | 35% | 95% |
| 20% | 40% | 91% |
| 25% | 45% | 88% |
| 30% | 50% | 86% |
| 40% | 60% | 83% |

The initial treatment benefit ("success" rate at day 4 of assessment period) is a key secondary end-point. The sample size of 107 patients per arm will also provide 80% power to detect an increase in the initial treatment benefit from 40% on placebo (this is a plausible maximum figure: a previous pilot study[3] of 19 patients observed a success rate of 26% [90% confidence interval 8%-42%] at a similar time point) to 60% on s-ketamine at the 5% 2-sided level of statistical significance using the chi-squared test. This sample size also means that the minimum power to detect an absolute improvement of 20% in initial treatment benefit is 80% whatever the placebo "success" rate.

A difference of 20% in the success rates is clearly clinically important and is the magnitude of effect that would be expected between the ketamine arm and placebo based on the pilot data.

As patients who drop-out of the study early (either during the titration or assessment period) will be treated as "failures" there is no need to recruit extra patients to compensate for this.

### 7.2 Analytical Plan
All analyses will be completed before the study code is broken. All analyses will be conducted on an intention-to-treat basis.

#### *7.2.1 Primary efficacy analysis*
The primary comparison will be in terms of time to treatment "failure" (as defined in 5.1.1) between the study arms. This comparison will be made using the log-rank test. The differences in the "success" rates between the arms at the day 16 assessment point will be estimated and presented together with associated 95% confidence intervals.

#### *7.2.2 Secondary efficacy analysis*
Initial benefit will be assessed by comparing the "success rates" at 4 days between the treatment arms using the chi-square test. The differences in the "success" rates will be estimated and presented together with associated 95 confidence intervals.

The analysis of VAS pain score will be complicated by missing data. Analysis of this data will use AUC techniques (Qian W, Parmar MKB, Sambrook RJ, Fayers PM, Girling DJ, Stephens RJ, Analysis of messy longitudinal data from a randomised clinical trial. Statistics in Medicine. 2000;2657-2647) coupled with multiple imputation.

The average opioid requirement will be compared between the treatment arms in terms of the morphine equivalent daily dose per day. This is measured over the titration and assessment periods.

The analysis of HADS (Hospital Anxiety and Depression Score), Quality of Life (EuroQol thermometer), LANSS the NCCN Distress Thermometer, the BTPQ and QST data will use the same approach as for VAS above.

#### *7.2.4 Safety Analysis*
The worst recorded toxicity grade for each patient (graded using NCI CTC AE v3.0) will be summarised by treatment arm and compared using the Mann-Whitney U-test.

#### *7.2.5 Interim Analysis*
The study data will be reviewed annually by a DMEC, primarily from a safety standpoint. There will be one formal interim analysis for efficacy after half the patients have been assessed. A p-value <.001 in the treatment comparison will trigger consideration of study closure or modification by the DMEC.

## 8 REGULATORY ISSUES

### 8.1 Clinical Trials Authorisation (CTA)
Cancer Research UK Clinical Trials Unit, Glasgow will apply to the MHRA for a clinical trials authorisation (CTA) to conduct the trial and will also be responsible for the maintenance of the CTA.

### 8.2 Ethics Approval
Ethical approval will be sought from a Main REC prior to commencement of this trial. The Local Research Ethics Committee (LREC) must approve each site before patients are entered at that site (Site Specific information SSI)

The study will be carried out in accordance with ICH GCP and the World Medical Association Declaration of Helsinki (1964) and its' revisions (Tokyo 1978, Venice 1983, Hong Kong 1989, South Africa 1996 and Edinburgh 2000).

### 8.3 Informed Consent
Consent to enter the study must be sought from each participant only after a full explanation has been given, the participant has been given an information sheet and time allowed for consideration. Signed participant consent should be obtained, the consent forms should also be signed by the person undertaking the consent procedure at site, who must be detailed on the site contact and responsibility log as having this authorisation. The Principal Investigator is responsible for ensuring if taking consent is delegated to a designee, the designee is suitably qualified by training or experience to take informed consent.

The right of the participant to refuse to participate without giving reasons must be respected. After the participant has entered the trial the clinician remains free to give alternative treatment to that specified in the protocol at any stage if he/she feels it is in the best interests of the participant, but the reasons for doing so must be recorded. In these cases the participants remain within the study for the purposes of follow-up and data analysis. All participants are free to withdraw at any time from the protocol treatment without giving reasons and without prejudicing further treatment.

Completed consent forms must be retained at each site, with one copy placed in the patient's casenote and another in the appropriate section of the Investigator Site File. All patients must be given a copy of the signed patient information sheet and consent form for their records. Consent forms must be retained on site and not submitted to the Trials Office.

In the event that new patient information sheets/consent forms are produced throughout the duration of the study, it maybe that patients already participating in the study should be re-consented to the updated version of the patient information sheet. However, if the principal investigator decides that this is not in the best interests of the patient re-consent is not required.

### 8.4 Patient Confidentiality

National Health Service Guidelines for storage, transmittal and disclosure of patient information will be followed at all times. Data on patients treated in the course of the study will be documented anonymously, that is patients will be identified only by a patient number and initials.

This study will be carried out to GCP Guidelines. Following formal admission to the study, patient data will be recorded in the hospital case records in the usual way including the circumstances of their entry into the study. Additionally data will be held in hard copy study case report form (CRF). These files will be identified by a study number, date of birth and patient initials only.

Representatives from the Study Sponsors and from the regulatory authorities will be given access to the records that relate to the study. They will have full access to all trial data as required.

Results of the study may be communicated at scientific meetings and will contribute to the scientific literature. At no time will this be done in such a way that an individual patient may be identified.

### 8.5 Liability, Indemnity and Insurance

The Hospital Trust at each participating site is responsible for the following:

1. Acts and omissions of its own staff and others engaged by it, including the Clinical Trials Unit and PI;
2. Ensuring the appropriate insurance administered by the National Health Service Litigation Authority is in place;
3. Ensuring any non-NHS employees involved in the clinical trial have Honorary Contracts with the Trust to cover access to patients and liability arrangements.

These responsibilities are outlined and agreed within the Clinical Study Agreement.

No special insurance is in place for patients in this study other than standard NHS liability insurance providing indemnity against clinical negligence. This does not provide cover for non-negligence e.g. harm caused by an unexpected side effect of participating in a study.

### 8.6 Funding

This study is being funded by a grant from Cancer Research UK (CTAAC).

### 8.7 Monitoring, Audits and Inspections

Trial Investigators must permit trial related monitoring, audits, REC review and regulatory inspections as required, by providing direct access to source data, CRFs and other documents (patients notes, trial site file, and other pertinent data).

A monitoring plan specific to this study is held separately to the protocol. The monitoring plan outlines the level of monitoring to be performed for the trial and how this will be carried out. The level of monitoring is based on the phase of the trial and any perceived risks identified by the risk assessment. Monitoring visits will commence after the Sponsor approves the plan.

It is understood that the CRUK Clinical Trial Monitor (or designee) will contact and visit participating Investigators regularly and will be allowed on request to inspect the various

records for the trial provided that patient confidentiality is maintained in accordance with local requirements**.**

The study may be subject to inspection and audit by NHS Greater Glasgow and Clyde/University of Glasgow under their remit as co-sponsors.

## 9 TRIAL MANAGEMENT AND DATA COLLECTION

### 9.1 Data Collection

Case report forms (CRFs) will be supplied by the Cancer Research UK Clinical Trials Unit, Glasgow. These forms should be completed in accordance with the CRF completion guidelines issued for the study. Queries should be handled as described in the study data-flow section of the CRF completion guidelines. Specific questions about data management should be addressed to the Clinical Trial Co-ordinator for the study:-

**Diann Taggart**
**Clinical Trial Co-ordinator**
**Cancer Research UK Clinical Trials Unit, Level 0**
**The Beatson West Of Scotland Cancer Centre**
**1053 Great Western Road**
**Glasgow G12 0YN**

**Tel:** **0141 301 7234**
**Fax:** **0141 301 7228**
**e-mail:** **diann.taggart@glasgow.ac.uk**

All CRFs must be returned to the Cancer Research UK Clinical Trials Unit, Glasgow for data entry and ultimately, statistical analysis.

CRFs for the study will be returned and stored in line with current regulatory requirements. Other essential documents, including source data, consent forms, and regulatory documents, will be archived by or for the Investigator in an appropriate archive facility in line with current regulatory requirements and made available for monitoring, audit and regulatory inspection.

### 9.2 Trial Management Group

A Trial Management Group (TMG) will oversee the running of the trial. Members of the TMG will include the Chief Investigator, Principal Investigator, Project Manager, Clinical Trial Co-ordinators, Trial Statistician, IT Staff, Quality Assurance Manager and Clinical Trial Monitor.

### 9.3 Trial Steering Committee

A Trial Steering Committee (TSC) will provide overall supervision for the trial. The TSC will be responsible for monitoring the progress of the trial towards its interim and overall objectives, focusing on adherence to the protocol, Good Clinical Practice (GCP), and patient safety. The TSC will include independent members who are not directly involved in other aspects of the trial.

### 9.4 Data Monitoring and Ethics Committee

A Data Monitoring Committee (DMEC) for the study will be set up to confidentially review the results of interim analyses. It will consist of two clinicians not directly involved in the study, but knowledgeable both of this therapeutic area and trial methodology, and an experienced clinical trials statistician. The study statistician will provide a detailed report to this Committee. The Committee will meet approximately annually and will review the information with particular reference to patient safety.

## 10 PUBLICATION AND AUTHORSHIP

The author names on any publication will include the protocol author, study co-ordinators, lead clinicians of each centre and the statistician. In addition a contributing institution may add a name of another clinician if this individual is responsible for > 10% of the total number of evaluable patients entered through that centre.

Trial data and results will be disseminated through various forums. Trial results will be published in peer-reviewed journals, which will achieve high impact. Trial findings will be made through oral presentations to learned societies and posters where appropriate.

No centre will publish data from an individual centre without prior approval of the Study Sponsor.

## 11 QUALITY ASSURANCE

Quality Assurance will be maintained by the following requirements and activities:

- Trial Investigators and Site staff must ensure that the trial is conducted in compliance with the protocol, Good Clinical Practice (GCP) and the applicable regulatory requirements.
- The Cancer Research UK Clinical Trials Unit, Glasgow (CTU) will assist the Trial Investigators and check they are complying with the protocol, GCP and regulatory requirements by monitoring trial documentation. Trial data and documentation will be checked for completeness, accuracy and reliability at monitoring visits.
- Central monitoring of trial data will be performed by the Trial Statistician and Clinical Trial Coordinator.
- The CTU will control data consistency and data quality by entering trial data onto the CTU trial database. Computerised and manual consistency checks will be performed and queries issued in cases of inconsistency or missing information. A full audit trail of any changes to the database will be maintained.
- An independent Data Monitoring and Ethics Committee (DMEC) will be established to oversee the safety and interim efficacy of the trial and will report their findings and recommendations to the Trial Steering Committee (TSC) and Trial Management Group (TMG) for implementation. The complete DMEC reports will remain confidential to the DMEC members and trial statistician.
- The TSC will ensure the trial is being managed effectively by the TMG.
- The TMG will ensure the trial is being managed according to the protocol, GCP and regulatory requirements on time and within budget.

## 12 ALLOCATION OF STUDY RESPONSIBILITIES

The co-sponsors of this clinical trial are NHS Greater Glasgow and Clyde and University of Glasgow.

Prior to study initiation, a non-commercially funded clinical trial co-sponsorship agreement will be put in place between NHS Greater Glasgow and Clyde and University of Glasgow. The role and liabilities each organisation will take are laid out in the agreement signed by both organisations. The University of Glasgow shall be responsible for carrying out the obligations and responsibilities set out in the aforementioned agreement, and shall be deemed the "sponsor" for the purposes of Part 3 of the Regulations in relation to the Study. NHS Greater Glasgow and Clyde shall be responsible for carrying out the obligations and responsibilities set out in the agreement, and shall be deemed the "sponsor" for the purposes of Parts 4, 5, 6 and 7 of the Regulations in relation to the Study.

A Clinical Study Agreement will be put in place between NHS Greater Glasgow and Clyde and each of the participating sites. This agreement outlines the responsibilities of each party's responsibilities in the running of the trial as well as the Chief Investigator (CI), the Cancer Research UK Clinical Trials Unit, Glasgow (CTU), and the Principal Investigator (PI) at the Participating Site.

### 12.1 Co-Sponsor Responsibilities (NHS Greater Glasgow and Clyde/University of Glasgow)

The Co-sponsor responsibilities will be for Authorisation and Ethics Committee opinion, GCP and Conduct and Pharmacovigilance. The majority of the Sponsor's responsibilities have been delegated to the Chief Investigator and these are performed by the CTU as the co-ordinating centre for the study. As such, the main role of the Sponsor is to ensure that the CTU fulfil their responsibilities as outlined in the Clinical Study Agreement and to ensure that any identified "risks" either have controls or action points put in place.

### 12.2 Clinical Trials Unit (CTU)

The CTU is responsible for the overall management of the clinical trial. This includes all regulatory submissions (ethics, R & D and CTA), all administration relating to the submissions, circulation of all correspondence to participating sites, data management, monitoring of data quality and safety, ongoing communication with participating sites, management of SAE/SUSAR reporting, and where applicable the management of any financial arrangements.

### 12.3 Chief Investigator (CI)

The Chief Investigator has delegated the majority of his/her responsibilities to the CTU. The CI is directly responsible for ensuring the protocol and any amendments are in place, for review of all SAEs and determination of whether they meet the criteria for a SUSAR, and to provide advice and recommendations on medical issues that arise involving the management of the patients on the study.

### 12.4 Participating Site

The Participating Site is responsible for the management of the trial within their site. This includes ensuring local ethical and management approval has been given, ensuring the study is conducted according to ICH GCP requirements, and ensuring the appropriate insurance or indemnity is in place. The Participating Site is also responsible for arranging access for on-site monitoring as identified in the study protocol for its site.

### 12.5 Principal Investigator (PI)

The PI is responsible for the delegation of study activities within their unit and ensuring all personnel are adequately trained and qualified to carry out their responsibilities. Regarding the management of patients within their site, the P.I. is responsible for the safety and well being of trial patients, reporting any deviations from the protocol to the CTU as well as any SAEs or safety issues.

Full details of the responsibilities are outlined in the Clinical Study Agreement. Two original copies of this will be held – one with the Sponsor and the other at the Participating Site. A photocopy of the signed agreement will also be held within the CTU.

## 13 DEFINITION OF END OF STUDY

For the purposes of Clinical Trial Authorisation the trial is deemed to have ended 30 days after the last patient remaining on treatment receives the last dose of ketamine or Placebo.

For the purposes of the Main REC approval, the study end date is deemed to be the date of the last data capture.

## 14 REFERENCES

1. Grond S, Zech D, Diefenbach C, Radburch L, Lehman KA. Assessment of cancer pain: a prospective evaluation in 2266 cancer patients referred to a pain service. Pain 1996;64:107-114

2. Caraceni A, Portenoy RK. An international survey of cancer pain characteristics and syndromes. Pain 1999;82:263-274

3. Glover J, Dibble SL, Dodd M, Miaskowski C. Mood states of oncology outpatients: does pain make a difference? JPSM 1995;10(2):120-128

4. Lloyd-Williams M, Dennis M, Taylor F. A prospective study to determine the association between physical symptoms and depression in patients with advanced cancer. Pall Med 2004;18:558-563

5.Mercadante S, Arccuri E, Tirelli W, Casuccio A. Analgesic effect of intravenous ketamine in cancer patients on morphine therapy: A randomised, controlled, double-blind, crossover, double-dose study. JPSM 2000;20:246-252

6. Mitchell AC. Fallon MT. A single infusion of intravenous ketamine improves pain relief in patients with critical limb ischaemia: results of a double blind randomised controlled trial. Pain 2002;97:275-281

7. Fallon MT. A randomised, double blind, placebo-controlled, parallel group comparative study of efficacy and tolerability of orally administered racemic ketamine and single enantiomer S-ketamine in the treatment of moderate to severe cancer-related neuropathic pain. Pilot Study. 2005. Submitted to EAPC Forum 2008

8. Bell RF, Eccleston C, Kalso E. Ketamine as an adjuvant to opioids for cancer pain. A Qualitative Systematic Review. JPSM 2003;26:867-867-875.

9. Elia N, Tramer MR. Ketamine and postoperative pain-a quantitative review systematic review of randomised trials. Pain 2005:113;61-70

10.Subramaniam K, Subramaniam B, Steinbrook RA. Ketamine as an adjuvant analgesic to opioids: A quantitative and qualitative systematic review. Anaesth Analog 2004:99:482-95

11. Fallon MT, Fergus C. Ketamine and other NMDA receptor antagonists In: Bennett M, editor. Oxford Pain Management Library, Neuropathic Pain. Oxford: Oxford University Press; 2006. p97-104.

12. Melzack R. The short form McGill Pain Questionnaire. Pain 1987 Aug;30(2):191-7.

13. Littman GS. Walker BR, Schneider BE. Reassessment of verbal and visualization analog ratings in analgesic studies. Clinical Pharmacology and Therapeutics.1985;38:16-23

14. Sriwatanakul K, Lasagna L, Cox C. Evaluation of current clinical trial methodology in analgesimetry based on experts opinions and analysis of several analgesic studies. Clinical Pharmacology and Therapeutics. 1983;34:277-283

15. Bennet MI. The LANSS Pain Scale: the Leeds assessment of neuropathic symptoms and signs. Pain 2001;92(1-2):147-157

16. Potter J, Higginson IJ, Scadding JW, Quigley CW. Identifying neuropathic pain in patients with head and neck cancer: use of the Leeds Assessment of Neuropathic Symptoms and Signs Scale. J R Soc Med 2003;96:379-83

17.Yucel A, Senocak M, Kocasoy Orhan E, Cimen A, Ertas M. Results of the Leeds Assessment of Neuropathic Symptoms and Signs Scale in Turkey: a validation study. J Pain 2004;5(8):427-32

18. Kaki AM, El-Yaski AZ, Youseif E. Identifying neuropathic pain among patients with chronic low-back pain: use of the Leeds Assessment of Neuropathic Symptoms and Signs Scale. Reg Anesth Pain Med 2005;30(5):422-8

19. Ransom S, Jacobsen PB, Booth-Jones M. Validation of the Distress Thermometer with bone marrow transplant patients. Psycho-oncology 2006 Jul;15(7):604-1221.

20. Patrick-Miller LJ, Broccoli TL, Much JK, Levine E. Validation of the Distress Thermometer: A single item screen to detect clinically significant psychological distress in ambulatory oncology patients. J Clin Oncol (Meeting Abstracts). 2004;22(14_suppl):6024.

21. Brooks R. EuroQol Group. EuroQol: the current state of play. Health Policy 1996; 37:53–72

22. Le Fevre P, Devereux J, Smith S, Lawrie SM, Cornbleet M. Screening for psychiatric illness in the palliative care inpatient setting: a comparison between the Hospital Anxiety and Depression Scale and the General Health Questionnaire-12. Pall Med 1999;13:399-407

23. Broadley KE, Kurowska A, Tookman A. Ketamine injection used orally. Pall Med 1996;10:247-250

24. Enarson M, Hays H, Woodroffe MA. Clinical experience with oral ketamine. JPSM 1999;17(5)384-386

25. Kisher K, Hagen N. Analgesic effect of oral ketamine in chronic neuropathic pain of spinal origin: a case report. JPSM 1999;18(1):61-66

## APPENDIX 1: OPIOD TITRATION SCHEDULE

The purpose of the "run-in period" is to stabilise the opioid dose prior to entry into the titration period. Each patient's opioid, opioid dose and pain will be different and thus titration will be done on an individual basis.

Opioid dose will be increased until **stable.** **Stable regimen defined as the same dose of controlled release and no more variation than 2 breakthrough opioid doses over the normal for that patient for a period of 2 days: two days without a change in opioid dose is sufficient to consider the opioid dose to be stable).**

During the run in period contact will be made with the patient on a daily basis.

At each point of contact, analgesia, side effects, breakthrough and total opioid requirements will be assessed. Titration will depend on the opioid preparation being used.

---

### Normal Release Preparations

Calculate the total amount of opioid required in the previous 24 hours.

Divide the total amount by six.

Prescribe this dose every four hours and alter the breakthrough dose (which will be one-sixth of the total daily dose OR the same as the four hourly dose)

If the patient is unable or unwilling to use breakthrough doses and pain control is still poor, the dose of normal release opioid should be increased, by 50%.

---

### Controlled Release Preparations

Calculate the total amount of opioid required in the previous 24 hours (i.e. controlled release preparations and breakthrough requirements)

Divide this total amount by 2.

Prescribe this amount twice daily.

Breakthrough dose will be one-sixth of the total amount.

If the patient is unable or unwilling to use breakthrough doses and pain control is still poor, the dose of controlled release opioid should be increased, by 50%.

---

Continue until opioid dose is stable. (**See definition of stable above**).

**All of the above is based on the recommendations of Scottish Intercollegiate Guidelines Network – Control of Pain in Patients with Cancer. SIGN 44.**

## APPENDIX 2: THE LANSS PAIN SCALE

The use of the LANSS[17] is a validated tool [18,19,20] allows an exact clarification of neuropathic pain for the purposes of this study. Whilst previous studies have shown palliative care staff to be very good at identifying patients with neuropathic pain, the use of the LANSS will add robustness to the study.

### THE LANSS PAIN SCALE

Leeds Assessment of Neuropathic Symptoms and Signs Pain Scale

**Initials (forename, surname): ___ ___ Today's Date:___/____/_____**

This pain scale can help to determine whether the nerves that are carrying your pain signals are working normally or not. It is important to find out in case different treatments are needed to control your pain.

### A. PAIN QUESTIONNAIRE (to be completed by patient)

- Think about how your pain has felt over the last week.
- Please say whether any of the descriptions match your pain exactly by ticking either the NO or YES box for each question.

**1) Does your pain feel like strange, unpleasant sensations in your skin? Words like prickling, tingling, pins and needles might describe these sensations.**

a) NO – My pain doesn't really feel like this ………………………………………....... ☐ (0)

b) YES – I get these sensations quite a lot ……………………………………….... ☐ (5)

**2) Does your pain make the skin in the painful area look different than normal? Words like mottled or looking more red or pink might describe the appearance.**

a) NO – My pain doesn't affect the colour of my skin …………………………………… ☐ (0)

b) YES – I've noticed that the pain does make my skin look different from normal …. ☐ (5)

**3) Does your pain make the affected skin abnormally sensitive to touch? Getting unpleasant sensations when lightly stroking the skin, or getting pain when wearing tight clothes might describe the abnormal sensitivity.**

a) NO – My pain doesn't make my skin abnormally sensitive in that area …………. ☐ (0)

b) YES – My skin seems abnormally sensitive to touch in that area ………………….. ☐ (3)

**4) Does your pain come on suddenly and in bursts for no apparent reason when you're still? Words like electric shocks, jumping and bursting describe these sensations.**

a) NO – My pain doesn't really feel like this ……………………………………………… ☐ (0)

b) YES – I get these sensations quite a lot ……………………………………………… ☐ (2)

**5) Does your pain feel as if the skin temperature in the painful area has changed abnormally? Words like hot and burning describe these sensations.**

a) NO – I don't really get these sensations ……………………………………………… ☐ (0)

b) YES – I get these sensations quite a lot ……………………………………………… ☐ (1)

## B. SENSORY TESTING (to be completed by Clinician)

Skin sensitivity can be examined by comparing the painful area with a contra lateral or adjacent non-painful area for the presence of allodynia and an altered pin-prick threshold (PPT).

**1) ALLODYNIA**

Examine the response to lightly stroking cotton wool across the non-painful area and then the painful area. If normal sensations are experienced in the non-painful site, but pain or unpleasant sensations (tingling, nausea) are experienced in the painful area when stroking, allodynia is present.

a) NO – normal sensation in both areas...................................................... ☐ (0)

b) YES – allodynia in painful area only...................................................... ☐ (5)

**2) ALTERED PIN-PRICK THRESHOLD**

Determine the pin-prick threshold by comparing the response to a 23 gauge (blue) needle mounted inside a 2 ml syringe barrel placed gently on the skin in a non-painful and then painful areas.

If a sharp needle prick is felt in the non-painful are, but a different sensation is experienced in the painful area e.g. non/blunt only (raised PPT) or a very painful sensation (lowered PPT), an altered PPT is present.

If a pinprick is not felt in either area, mount the syringe onto the needle to increase the weight and repeat.

a) NO – equal sensation in both areas ...................................................... ☐ (0)

b) YES – altered PPT in painful area ...................................................... ☐ (3)

---

SCORING:

Add values in parentheses for sensory description and examination findings to obtain overall score.

TOTAL SCORE (maximum 24).......................................................................... ☐

If score <12, neuropathic mechanisms are **unlikely** to be contributing to the patient's pain
If score >12, neuropathic mechanisms are **likely** to be contributing to the patient's pain

**APPENDIX 3: VISUAL ANALOGUE SCORE (VAS)**

Scoring tools such as visual analogue scales for pain intensity and relief are more sensitive than pain category scales in detecting drug treatment effects.[21,22]

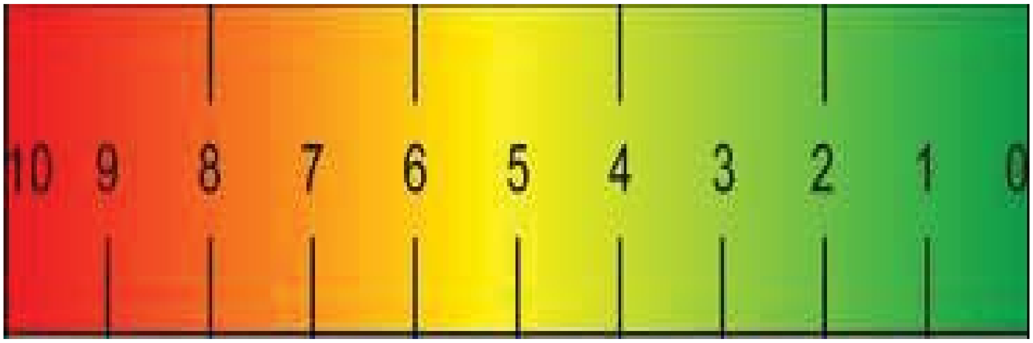


**Worst pain** **No Pain**

## APPENDIX 4: MCGILL SHORT FORM PAIN QUESTIONNAIRE

This abbreviated version of the McGill Pain Questionnaire (SF-MPQ)[23] consists of 15 descriptors (11 sensory; 4 affective) which are rated on an intensity scale as 0 = none, 1 = mild, 2 = moderate or 3 = severe. In this study only the sensory component of the SF-MPQ are used as these are specific for neuropathic pain. This validated tool is useful in this situation where the standard MPQ would take a considerable time to administer yet data is desirable.

### I. Pain Rating Index (PRI):

These words below describe pain in the past 24 hours. Please place a check mark (√) in the column that represents the degree to which you feel that type of pain.

| | | None | | Mild | | Moderate | | Severe |
|---|---|---|---|---|---|---|---|---|
| **Throbbing** | 0 | | 1 | | 2 | | 3 | |
| **Shooting** | 0 | | 1 | | 2 | | 3 | |
| **Stabbing** | 0 | | 1 | | 2 | | 3 | |
| **Sharp** | 0 | | 1 | | 2 | | 3 | |
| **Cramping** | 0 | | 1 | | 2 | | 3 | |
| **Gnawing** | 0 | | 1 | | 2 | | 3 | |
| **Hot-Burning** | 0 | | 1 | | 2 | | 3 | |
| **Aching** | 0 | | 1 | | 2 | | 3 | |
| **Heavy** | 0 | | 1 | | 2 | | 3 | |
| **Tender** | 0 | | 1 | | 2 | | 3 | |
| **Splitting** | 0 | | 1 | | 2 | | 3 | |

## APPENDIX 5: NCCN DISTRESS THERMOMETER

This is another rating score similar to the visual analogue score used for measuring pain: 0 (no distress) to 10 (extreme distress). The single item questionnaire has been validated and is a quick and reliable tool which will identify distress.[24,25] The patient places a mark on the scale answering: "How distressed have you been during the past week on a scale of 0 to 10?" Scores of 4 or more indicate a significant level of distress that will be evaluated.

**Instructions: First please circle the number (0-10) that best describes how much distress you have been experiencing in the past week including today.**

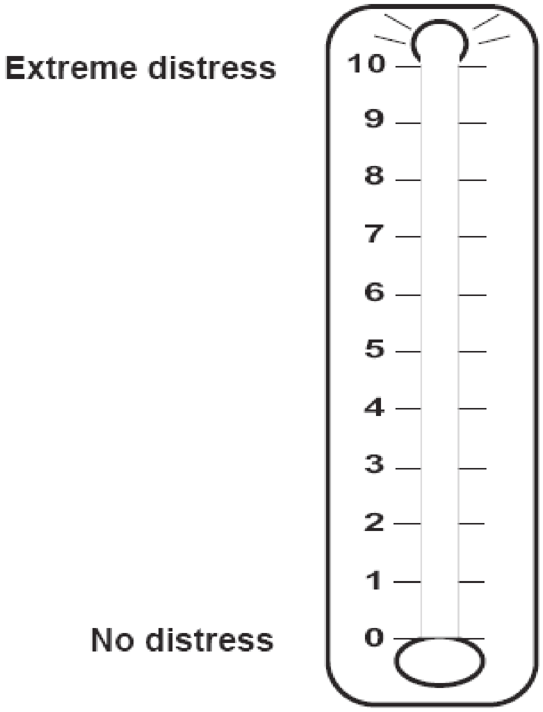

## APPENDIX 6: HOSPITAL ANXIETY AND DEPRESSION SCALE

This 14 question score has been used extensively in cancer studies and validated thus.[26] It can be completed either by the patient or with the assistance of trial staff. A combined score of 20 or more suggests a diagnosis of depression.

**Instructions:**

This questionnaire is designed to assess how you feel. Read each item and tick the box of the reply which comes closest to how you have been feeling **in the past week.** Please tick only one reply for each item.

Don't take too long over your replies: your immediate reaction to each item will probably be more accurate than a long thought out response.

| | | | |
|---|---|---|---|
| **I feel tense or 'wound up':** | | **I feel as if I am slowed down:** | |
| Most of the time | | Nearly all of the time | |
| A lot of the time | | Very often | |
| Time to time, occasionally | | Sometimes | |
| Not at all | | Not at all | |
| **I still enjoy the things I used to enjoy:** | | **I get a sort of frightened feeling like 'butterflies in the stomach':** | |
| Definitely as much | | Not at all | |
| Not quite so much | | Occasionally | |
| Only a little | | Quite often | |
| Not at all | | Very often | |
| **I get a sort of frightened feeling like something awful is about to happen:** | | **I have lost interest in my appearance:** | |
| Very definitely and quite badly | | Definitely | |
| Yes, but not too badly | | I don't take as much care as I should | |
| A little, but it doesn't worry me | | I may not take quite as much care | |
| Not at all | | I take just as much care as ever | |
| **I can laugh and see the funny side of things:** | | **I feel restless as if I have to be on the move:** | |
| As much as I always could | | Very much indeed | |
| Not quite so much now | | Quite a lot | |
| Definitely not so much now | | Not very much | |
| Not al all | | Not at all | |
| **Worrying thoughts go through my mind:** | | **I look forward with enjoyment to things:** | |
| A great deal of the time | | A much as I ever did | |
| A lot of the time | | Rather less than I used to | |
| From time to time but not too often | | Definitely less than I used to | |
| Only occasionally | | Hardly at all | |

**I feel cheerful:**

Not at all
Not often
Sometimes
Most of the time

**I get sudden feelings of panic:**

Very often indeed
Quite often
Not very often
Not at all

**I can sit at ease and feel relaxed:**

Definitely
Usually
Not often
Not at all

**I can enjoy a good book or radio or TV programme:**

Often
Sometimes
Not often
Very seldom

## APPENDIX 7: EUROQOL THERMOMETER

This is an abbreviated version of the Euroqol Quality of Life questionnaire and exists as a visual analogue score which is easy to understand and complete.[27]

Best possible quality of life

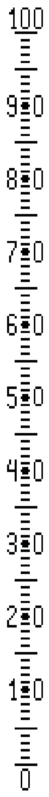


Worst possible quality of life

## APPENDIX 8: QST

**QST**

| | |
|---|---|
| Spontaneous pain VAS result (0-10) | |

Area with abnormal sensation: Yes ☐ No ☐ Map out area with marker.

| | Test Area | | Control Area | |
|---|---|---|---|---|
| | Result * | VAS | Result | VAS |
| Brush | | | | |
| Von Frey filaments | | | | |
| - detection threshold | | | | |
| - pain threshold | | | | |
| Rolltemp | | | | |
| - cool | | | | |
| - warm | | | | |
| Pin prick | | | | |
| Wind-up | | | | |

* increased / reduced / no difference than control area

## APPENDIX 9: BREAKTHROUGH PAIN QUESTIONNAIRE

### Breakthrough Pain Questionnaire

1. **Thinking about your usual or background pain, on average, how severe has this been in the last 24 hours?** (Please circle how severe the pain has been).

   **0 1 2 3 4 5 6 7 8 9 10**

2. **In the last 24 hours, how many times has your pain flared up or become severe?** (Please circle the number of times that you have had flare-ups of pain)

   **Not sure**

   **1 2 3 4 5 6 7 8 9 10** >10

3. **If more than 10, how many times:** ________ **Not sure**

4. **In the last 24 hours on average, how severe does the pain become when it flares up?** (Please circle how severe the pain has been).

   **0 1 2 3 4 5 6 7 8 9 10**

5. **Over the last 24 hours, on average, how long does the flare up of pain last for?** (Please circle the number of minutes)

   **(less than 1min) (1-15mins) (16-30mins) (31-60mins)**

   **(60-120mins) (more than 120 mins)**

6. **Over the last 24 hours, on average, from the time you first feel this pain start to flare-up or worsen, how long does it take to get as bad as it gets?** (Please tick the appropriate box)

   **Unpredictable** ☐ **6 minutes to 30 minutes** ☐

   **Less than 10 seconds** ☐ **31 minutes to 60 minutes** ☐

   **10 seconds to 5 minutes** ☐

7. **Are you able to tell or predict when your pain is going to flare-up or become severe?** (Please circle the phrase that most closely represents what you think)

   **never sometimes often almost always always**

8. **How often do you take extra pain killers or a "rescue" dose for your flare ups of pain?** (Please circle the response that you feel most accurately describes what normally happens)

   **every time most of the time some of the time hardly ever never**

BPQ Version 1 17th July 2009
Authors: Professor Marie Fallon
Dr Barry Laird

## APPENDIX 10: PHARMACOGENETIC BLOOD SAMPLING

Take two x 10mls venous samples and place separately into a Lithium Heparin and an EDTA container.

These should be sent to:

Lee Murphy (E090703)
Laboratory Manager
Genetics Core
Wellcome Trust Research Facility
Western General Hospital
EDINBURGH EH4 2XU

## APPENDIX 11: RUN OUT SCHEDULE

### Dose Level 7

| Day | Total Dose mg | 10mg capsules | 20mg capsules | 40mg capsules |
|---|---|---|---|---|
| 7 | 320 | | | 8 |
| 6 | 240 | | 4 | 4 |
| 5 | 240 | | 4 | 4 |
| 4 | 160 | | | 4 |
| 3 | 120 | 4 | 4 | |
| 2 | 80 | | 4 | |
| 1 | 40 | 4 | | |

### Dose Level 6

| Day | Total Dose mg | 10mg capsules | 20mg capsules | 40mg capsules |
|---|---|---|---|---|
| 7 | 240 | | 4 | 4 |
| 6 | 240 | | 4 | 4 |
| 5 | 160 | | | 4 |
| 4 | 160 | | | 4 |
| 3 | 120 | 4 | 4 | |
| 2 | 80 | | 4 | |
| 1 | 40 | 4 | | |

### Dose Level 5

| Day | Total Dose mg | 10mg capsules | 20mg capsules | 40mg capsules |
|---|---|---|---|---|
| 7 | 120 | 4 | 4 | |
| 6 | 120 | 4 | 4 | |
| 5 | 80 | | 4 | |
| 4 | 80 | | 4 | |
| 3 | 40 | 4 | | |
| 2 | 40 | 4 | | |
| 1 | 40 | 4 | | |

**Dose Level 4**

| Day | Total Dose mg | 10mg capsules | 20mg capsules | 40mg capsules |
|---|---|---|---|---|
| 7 | 120 | 4 | 4 | |
| 6 | 120 | 4 | 4 | |
| 5 | 80 | | 4 | |
| 4 | 80 | | 4 | |
| 3 | 40 | 4 | | |
| 2 | 40 | 4 | | |
| 1 | 40 | 4 | | |

**Dose Level 3**

| Day | Total Dose mg | 10mg capsules | 20mg capsules | 40mg capsules |
|---|---|---|---|---|
| 7 | 120 | 4 | 4 | |
| 6 | 120 | 4 | 4 | |
| 5 | 80 | | 4 | |
| 4 | 80 | | 4 | |
| 3 | 40 | 4 | | |
| 2 | 40 | 4 | | |
| 1 | 40 | 4 | | |
| | | 20 | 16 | |

**Dose Level 2**

| Day | Total Dose mg | 10mg capsules | 20mg capsules | 40mg capsules |
|---|---|---|---|---|
| 7 | 80 | | 4 | |
| 6 | 60 | 6 | | |
| 5 | 60 | 6 | | |
| 4 | 30 | 3 | | |
| 3 | 30 | 3 | | |
| 2 | 20 | 2 | | |
| 1 | 10 | 1 | | |

**Dose Level 1**

| Day | Total Dose mg | 10mg capsules | 20mg capsules | 40mg capsules |
|---|---|---|---|---|
| 7 | 40 | 4 | | |
| 6 | 40 | 4 | | |
| 5 | 30 | 3 | | |
| 4 | 30 | 3 | | |
| 3 | 20 | 2 | | |
| 2 | 20 | 2 | | |
| 1 | 10 | 1 | | |

## APPENDIX 12: DECLARATION OF HELSINKI 2000

## DECLARATION OF HELSINKI
## WORLD MEDICAL ASSOCIATION DECLARATION OF HELSINKI

### Ethical Principles for Medical Research Involving Human Subjects

Adopted by the 18th WMA General Assembly
Helsinki, Finland, June 1964
and amended by the
29th WMA General Assembly, Tokyo, Japan, October 1975
35th WMA General Assembly, Venice, Italy, October 1983
41st WMA General Assembly, Hong Kong, September 1989
48th WMA General Assembly, Somerset West, Republic of South Africa, October 1996
and the
52nd WMA General Assembly, Edinburgh, Scotland, October 2000

### A. INTRODUCTION

1. The World Medical Association has developed the Declaration of Helsinki as a statement of ethical principles to provide guidance to physicians and other participants in medical research involving human subjects. Medical research involving human subjects includes research on identifiable human material or identifiable data.

2. It is the duty of the physician to promote and safeguard the health of the people. The physician's knowledge and conscience are dedicated to the fulfilment of this duty.

3. The Declaration of Geneva of the World Medical Association binds the physician with the words, "The health of my patient will be my first consideration," and the International Code of Medical Ethics declares that, "A physician shall act only in the patient's interest when providing medical care which might have the effect of weakening the physical and mental condition of the patient."

4. Medical progress is based on research which ultimately must rest in part on experimentation involving human subjects.

5. In medical research on human subjects, considerations related to the well-being of the human subject should take precedence over the interests of science and society.

6. The primary purpose of medical research involving human subjects is to improve prophylactic, diagnostic and therapeutic procedures and the understanding of the etiology and pathogenesis of disease. Even the best proven prophylactic, diagnostic, and therapeutic methods must continuously be challenged through research for their effectiveness, efficiency, accessibility and quality.

7. In current medical practice and in medical research, most prophylactic, diagnostic and therapeutic procedures involve risks and burdens.

8. Medical research is subject to ethical standards that promote respect for all human beings and protect their health and rights. Some research populations are

vulnerable and need special protection. The particular needs of the economically and medically disadvantaged must be recognized. Special attention is also required for those who cannot give or refuse consent for themselves, for those who may be subject to giving consent under duress, for those who will not benefit personally from the research and for those for whom the research is combined with care.

9. Research Investigators should be aware of the ethical, legal and regulatory requirements for research on human subjects in their own countries as well as applicable international requirements. No national ethical, legal or regulatory requirement should be allowed to reduce or eliminate any of the protections for human subjects set forth in this Declaration.

## B. BASIC PRINCIPLES FOR ALL MEDICAL RESEARCH

10. It is the duty of the physician in medical research to protect the life, health, privacy, and dignity of the human subject.

11. Medical research involving human subjects must conform to generally accepted scientific principles, be based on a thorough knowledge of the scientific literature, other relevant sources of information, and on adequate laboratory and, where appropriate, animal experimentation.

12. Appropriate caution must be exercised in the conduct of research which may affect the environment, and the welfare of animals used for research must be respected.

13. The design and performance of each experimental procedure involving human subjects should be clearly formulated in an experimental protocol. This protocol should be submitted for consideration, comment, guidance, and where appropriate, approval to a specially appointed ethical review committee, which must be independent of the investigator, the sponsor or any other kind of undue influence. This independent committee should be in conformity with the laws and regulations of the country in which the research experiment is performed. The committee has the right to monitor ongoing trials. The researcher has the obligation to provide monitoring information to the committee, especially any serious adverse events. The researcher should also submit to the committee, for review, information regarding funding, sponsors, institutional affiliations, other potential conflicts of interest and incentives for subjects.

14. The research protocol should always contain a statement of the ethical considerations involved and should indicate that there is compliance with the principles enunciated in this Declaration.

15. Medical research involving human subjects should be conducted only by scientifically qualified persons and under the supervision of a clinically competent medical person. The responsibility for the human subject must always rest with a medically qualified person and never rest on the subject of the research, even though the subject has given consent.

16. Every medical research project involving human subjects should be preceded by careful assessment of predictable risks and burdens in comparison with foreseeable benefits to the subject or to others. This does not preclude the participation of healthy volunteers in medical research. The design of all studies should be publicly available.

17. Physicians should abstain from engaging in research projects involving human subjects unless they are confident that the risks involved have been adequately assessed and can be satisfactorily managed. Physicians should cease any investigation if the risks are found to outweigh the potential benefits or if there is conclusive proof of positive and beneficial results.

18. Medical research involving human subjects should only be conducted if the importance of the objective outweighs the inherent risks and burdens to the subject. This is especially important when the human subjects are healthy volunteers.

19. Medical research is only justified if there is a reasonable likelihood that the populations in which the research is carried out stand to benefit from the results of the research.

20. The subjects must be volunteers and informed participants in the research project.

21. The right of research subjects to safeguard their integrity must always be respected. Every precaution should be taken to respect the privacy of the subject, the confidentiality of the patient's information and to minimize the impact of the study on the subject's physical and mental integrity and on the personality of the subject.

22. In any research on human beings, each potential subject must be adequately informed of the aims, methods, sources of funding, any possible conflicts of interest, institutional affiliations of the researcher, the anticipated benefits and potential risks of the study and the discomfort it may entail. The subject should be informed of the right to abstain from participation in the study or to withdraw consent to participate at any time without reprisal. After ensuring that the subject has understood the information, the physician should then obtain the subject's freely-given informed consent, preferably in writing. If the consent cannot be obtained in writing, the non-written consent must be formally documented and witnessed.

23. When obtaining informed consent for the research project the physician should be particularly cautious if the subject is in a dependent relationship with the physician or may consent under duress. In that case the informed consent should be obtained by a well-informed physician who is not engaged in the investigation and who is completely independent of this relationship.

24. For a research subject who is legally incompetent, physically or mentally incapable of giving consent or is a legally incompetent minor, the investigator must obtain informed consent from the legally authorized representative in accordance with applicable law. These groups should not be included in research unless the research is necessary to promote the health of the population represented and this research cannot instead be performed on legally competent persons.

25. When a subject deemed legally incompetent, such as a minor child, is able to give assent to decisions about participation in research, the investigator must obtain that assent in addition to the consent of the legally authorized representative.

26. Research on individuals from whom it is not possible to obtain consent, including proxy or advance consent, should be done only if the physical/mental condition that prevents obtaining informed consent is a necessary characteristic of the research population. The specific reasons for involving research subjects

with a condition that renders them unable to give informed consent should be stated in the experimental protocol for consideration and approval of the review committee. The protocol should state that consent to remain in the research should be obtained as soon as possible from the individual or a legally authorized surrogate.

27. Both authors and publishers have ethical obligations. In publication of the results of research, the investigators are obliged to preserve the accuracy of the results. Negate as well as positive results should be published or otherwise publicly available. Sources of funding, institutional affiliations and any possible conflicts of interest should be declared in the publication. Reports of experimentation not in accordance with the principles laid down in this Declaration should not be accepted for publication.

## C. ADDITIONAL PRINCIPLES FOR MEDICAL RESEARCH COMBINED WITH MEDICAL CARE

28. The physician may combine medical research with medical care, only to the extent that the research is justified by its potential prophylactic, diagnostic or therapeutic value. When medical research is combined with medical care, additional standards apply to protect the patients who are research subjects.

29. The benefits, risks, burdens and effectiveness of a new method should be tested against those of the best current prophylactic, diagnostic, and therapeutic methods. This does not exclude the use of placebo, or no treatment, in studies where no proven prophylactic, diagnostic or therapeutic method exists.

30. At the conclusion of the study, every patient entered into the study should be assured of access to the best proven prophylactic, diagnostic and therapeutic methods identified by the study.

31. The physician should fully inform the patient which aspects of the care are related to the research. The refusal of a patient to participate in a study must never interfere with the patient-physician relationship.

32. In the treatment of a patient, where proven prophylactic, diagnostic and therapeutic methods do not exist or have been ineffective, the physician, with informed consent from the patient, must be free to use unproven or new prophylactic, diagnostic and therapeutic measures, if in the physician's judgment it offers hope of saving life, re-establishing health or alleviating suffering. Where possible, these measures should be made the object of research, designed to evaluate their safety and efficacy. In all cases, new information should be recorded and, where appropriate, published. The other relevant guidelines of this Declaration should be followed.

# STATISTICAL ANALYSIS PLAN

**Protocol Number :** MI69

**Protocol Title :** KPS (KETAMINE IN PAIN STUDY)
A RANDOMISED DOUBLE-BLIND CONTROLLED TRIAL OF KETAMINE VERSUS PLACEBO IN CONJUNCTION WITH BEST PAIN MANAGEMENT IN NEUROPATHIC PAIN IN CANCER PATIENTS

**Author:** Caroline Bray
Senior Statistician
Cancer Research UK Clinical Trials Unit, Glasgow
(partner in CaCTUS – Cancer Clinical Trials Unit Scotland)
The Beatson West of Scotland Cancer Centre Level 0
1053 Gt. Western Road
Glasgow, G12 0YN

**Version:** Version 3
**Amendments:** Updated sections: 4.1, 7.10.1

**Approval Date:** 4 January 2012

**Checked, approved and signed:**

| | | **Signature** | **Date** |
|---|---|---|---|
| **Statistician (author):** | Caroline Bray | | |
| **Statistician (reviewer):** | Jim Paul | | |
| **Chief Investigators:** | Marie Fallon | | |
| | Barry Laird | | |

## Contents


1 TRIAL OBJECTIVES ... 3
2 TRIAL DESIGN ... 3
2.1 Study Stages ... 3
3 SAMPLE SIZE CALCULATIONS ... 5
4 DEFINITION OF EFFICACY PARAMETERS ... 5
4.1 Primary efficacy parameters ... 5
4.2 Secondary efficacy parameters ... 6
4.3 Procedures for assessing efficacy parameters ... 6
5 DEFINITION OF SAFETY PARAMETERS ... 7
5.1 Adverse events ... 7
5.2 Serious adverse events and serious adverse drug reactions ... 7
5.3 Definition of a serious adverse event ... 7
5.4 Definition of Suspected Unexpected Serious Adverse Reactions ... 7
6 DEFINITION OF POPULATIONS ... 7
6.1 Intention-to-treat population ... 7
6.2 Eligible study population ... 7
6.3 Per protocol population ... 7
6.4 Run-in stage population ... 8
6.5 Safety population ... 8
7 TABULATIONS, FIGURES AND LISTINGS ... 8
7.1 Study Recruitment ... 8
7.2 Form Return ... 8
7.3 Patient progress ... 8
7.4 Eligibility/ population membership ... 8
7.4.1 Registration eligibility ... 8
7.4.2 Randomisation eligibility ... 8
7.4.3 Population membership ... 9
7.5 Baseline data and demographic characteristics ... 9
7.5.1 Trial accrual ... 9
7.5.2 Run-in period ... 9
7.5.3 Baseline data at randomisation ... 9
7.6 Delivery of study therapy ... 10
7.6.1 Titration period ... 10
7.6.2 Assessment period ... 10
7.6.3 Run-out period ... 11
7.7 Opioid Use ... 11
7.8 Unblinding ... 11
7.9 Safety analyses ... 11
7.9.1 Run-in period ... 11
7.9.2 Adverse events ... 11
7.9.3 Serious adverse events/reactions/SUSARs ... 12
7.10 Efficacy analyses ... 12
7.10.1 Primary efficacy analysis ... 12
7.10.2 Secondary efficacy analysis ... 12
7.11 Concomitant medication ... 13
8 STATISTICAL METHODS FOR THE ANALYSIS OF THE STUDY DATA ... 13
8.1 Primary efficacy analysis ... 13
8.2 Secondary efficacy analyses ... 13
8.3 Interim analyses ... 14
9 REFERENCES ... 14

# 1 TRIAL OBJECTIVES

The objectives of this randomised double-blind phase III trial are:

Primary: To establish whether ketamine given in addition to best standard pain management improves malignant neuropathic pain compared to best standard pain management alone. This is assessed using the sensory component of the Short Form McGill Pain Questionnaire (SF-MPQ).

Secondary:

- To compare initial treatment benefit (at day 4 of assessment period of 16 days) using the sensory component of the SF-MPQ.
- To compare difference in overall pain between the study arms based on the VAS score.
- To compare difference in neuropathic pain between the study arms based on the LANSS pain scale.
- To assess worst pain score (index neuropathic site) between the two arms.
- To compare patient distress between the two arms based on NCCN Distress Thermometer.
- To assess the side-effects and tolerability of trial drug.
- To assess the effect of intervention on quality of life scores (based on Euroqol thermometer), anxiety and depression (based on HAD scale) and opioid requirements.
- To assess the effect of intervention on sensory changes (as assessed by Quantitative Sensory Testing).
- To assess the effect of intervention on Breakthrough Cancer Pain (BTcP) using the Breakthrough Pain Questionnaire.

# 2 TRIAL DESIGN

This is a randomised double-blind trial comparing ketamine with placebo in patients with malignant neuropathic pain. Following a run-in period where opioid analgesia dose will be optimised, ketamine or placebo will be administered orally four times a day. The dose will be increased as per the titration schedule (Appendix I of protocol) and dose increments will cease when pain allows or before then if toxicity is unacceptable.

The trial will be carried out in 4 stages (run-in, titration, assessment and run-out).

The trial is stratified (using minimisation) for centre, age, baseline McGill sensory pain score, gender and type of previous adjuvant analgesic for neuropathic pain.

## 2.1 Study Stages

The trial will be carried out in 4 stages (see chart 1 on next page):

**Chart 1 – Trial Stages**

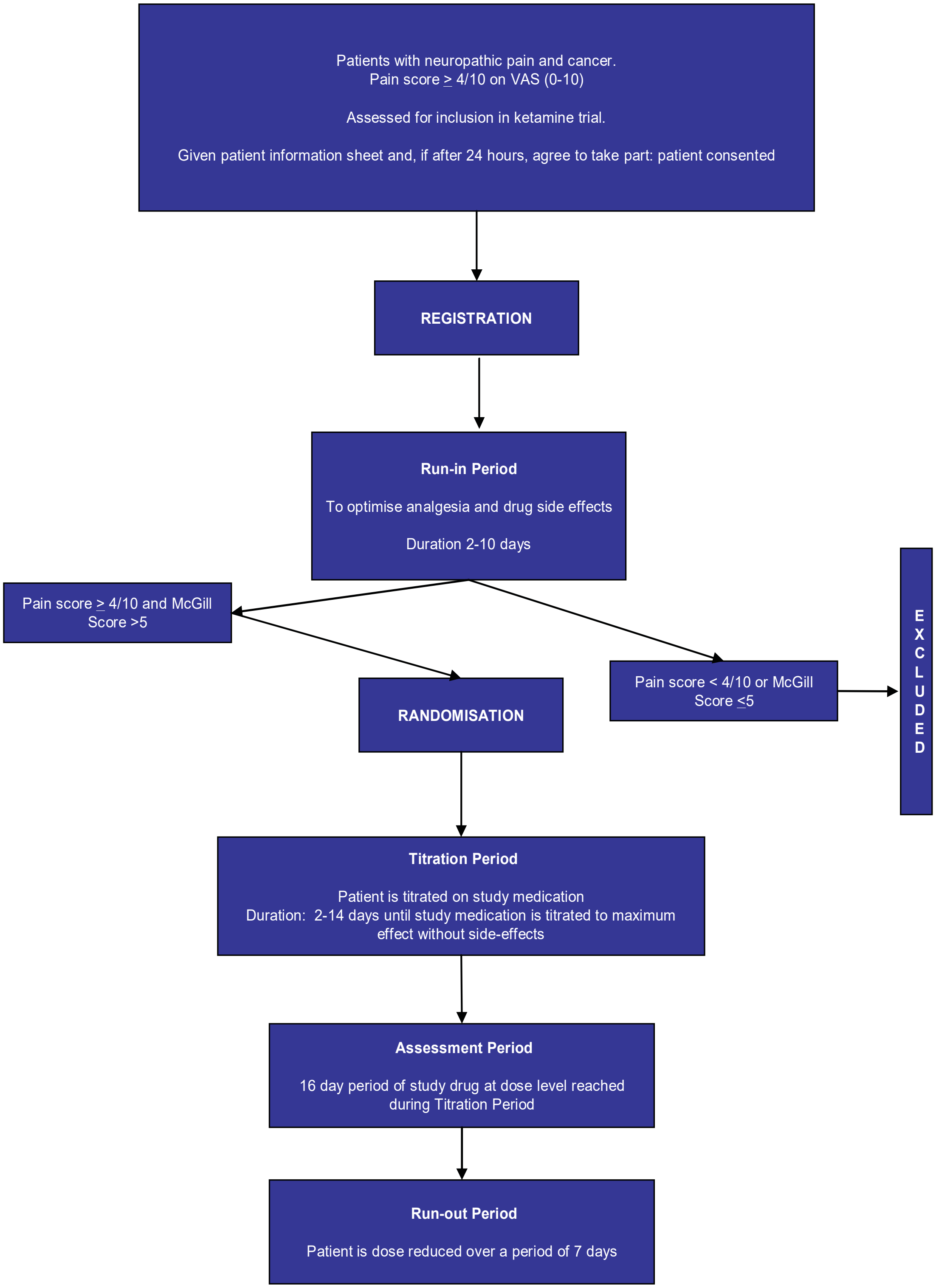

# 3 SAMPLE SIZE CALCULATIONS

The primary endpoint is improvement in malignant neuropathic (assessed using the sensory component of the SF-MPQ). A key secondary endpoint is the initial treatment benefit ("success" rate at day 4 of assessment period).

The proposed sample size of 107 patients per arm will provide at least 80% power to detect an increase in the "success" (patients who remain on study with no significant change in opioid dose and have a 5-point drop in the sensory component of the SF-MPQ.) rate at day 16 of 20% on Ketamine compared to placebo over a range of possible placebo "success rates". These figures are based on the logrank test and a 5% two-sided level of statistical significance.

| **Success rate at day 16 of assessment period** | | **Study Power** |
|---|---|---|
| Placebo | Ketamine | |
| 10% | 25% | 90% |
| 10% | 30% | 98% |
| 15% | 30% | 82% |
| 15% | 35% | 95% |
| 20% | 40% | 91% |
| 25% | 45% | 88% |
| 30% | 50% | 86% |
| 40% | 60% | 83% |

A sample size of 107 patients per arm will also provide 80% power to detect an increase in the initial treatment benefit from 40% on placebo to 60% on ketamine at the 5% 2-sided level of statistical significance using the chi-squared test. This sample size also means that the minimum power to detect an absolute improvement of 20% in initial treatment benefit is 80% whatever the placebo "success" rate.

As patients who drop-out of the trial early (either during the titration or assessment period) will be treated as "failures" there is no need to recruit extra patients to compensate for this.

# 4 DEFINITION OF EFFICACY PARAMETERS

## 4.1 Primary efficacy parameters

Efficacy of therapy will be evaluated by comparing the treated and control groups of patients in terms of the improvement in neuropathic pain as assessed using the sensory component of the SF-MPQ.

The primary trial end-point is time to treatment "failure".

A treatment "failure" is defined as

- failure to achieve a 5-point drop in the sensory component of the SF-MPQ, from the end of the run in period (prior to randomisation) to any one of the assessment time points (end of titration period, day 4, day 8, day 12, day 16)
- a significant change in prescribed background opioid dose (defined as greater than 30% increase in 24 hour morphine equivalent daily dose [MEDD] during titration or assessment period)

- a withdrawal from the trial during the titration or assessment period for any reason (e.g. lack of efficacy, side effects)

Conversely a treatment “success” is a patient who remains in the trial with no significant change in prescribed background opioid dose and has a 5-point drop in the sensory component of the SF-MPQ.

### 4.2 Secondary efficacy parameters

a) The initial treatment benefit (at day 4 of assessment period of 16 days) using the sensory component of the SF-MPQ.
b) The difference in overall pain between the study arms based on the VAS score.
c) The difference in neuropathic pain between the study arms based on the Leeds Assessment of Neuropathic Symptoms and Signs (LANSS) pain scale.
d) The worst pain score (index neuropathic site) in the previous 24 hours (between the two arms) at study baseline and then during study assessment period.
e) Patient distress between the two arms based on NCCN Distress Thermometer.
f) Side-effects and tolerability of trial drug.
g) The effect of the intervention on quality of life scores (based on Euroqol thermometer), anxiety and depression (based on HAD scale) and opioid requirements.
h) To assess the effect of intervention on sensory changes (as assessed by Quantitative Sensory Testing (QST)).
i) To assess the effect of intervention on Breakthrough Cancer Pain (BTcP) using the Breakthrough Pain Questionnaire.

### 4.3 Procedures for assessing efficacy parameters

The above tools are applied at the following time-points:-

- SF-MPQ (sensory component) Start of run in period, end of run in period (prior to randomisation), daily throughout titration and days 1, 4, 8, 12 and 16 of assessment period.
- VAS pain score – Daily throughout run in, titration and assessment period.
- LANSS – Start of run in period, end of run in period (prior to randomisation), and day 1, 4, 8, 12 and 16 of assessment period.
- Opioid use (24 hour oral morphine equianalgesic equivalent) – Start of run-in period, end of run-in period (prior to randomisation) and daily during titration and assessment periods.
- HADS – anxiety/depression - End of run in period (prior to randomisation), and day 1, 4, 8, 12 and 16 of assessment period.
- Euroqol thermometer – Quality of Life – End of run in period (prior to randomisation) and day 1, 4, 8, 12 and 16 of assessment period.
- Distress – using NCCN Distress Thermometer End of run in period (prior to randomisation) and day 1, 4, 8, 12 and 16 of assessment period.
- Breakthrough Cancer Pain – using the BTPQ End of run in period and day 16 of assessment period.
- Sensory testing – using QST End of run in period and day 16 of assessment period.

# 5 DEFINITION OF SAFETY PARAMETERS

## 5.1 Adverse events

All adverse events (AEs) occurring during the run-in, titration, assessment and run-out stages or up to 30 days after the administration of the last trial treatment for any individual patient will be graded (CTCAE v3.0) and recorded on the relevant CRF. An AE is any untoward medical occurrence or experience in a patient that occurs following the administration of the trial medication regardless of the dose or causal relationship.

## 5.2 Serious adverse events and serious adverse drug reactions

A serious adverse events (SAE) is defined as an event that:

- results in death
- is life-threatening (the patient was at immediate risk of death at the time reaction was observed)
- requires hospitalisation or prolongation of hospitalisation
- results in persistent or significant disability/incapacity
- is a congenital anomaly/birth defect
- is considered medically significant by the investigator

The assessment of causality is made by the investigator using the definitions in §6.4 of the protocol.

## 5.3 Definition of a serious adverse event

A serious adverse reaction (SAR) is an SAE that may be related to trial treatment. SAEs that will be considered related will include any SAE that is documented as possibly, probably or definitely related to protocol treatment.

## 5.4 Definition of Suspected Unexpected Serious Adverse Reactions

A suspected unexpected serious adverse reaction (SUSAR) is any serious adverse reaction that is unexpected. Unexpected is any reaction that is not a known reaction listed in the IMPD/Summary of Product Characteristics (SmPC).

# 6 DEFINITION OF POPULATIONS

As this trial has a pre-randomisation run-in period, the following analysis populations will be defined from all patients that are registered onto the study.

## 6.1 Intention-to-treat population

The intention-to-treat (ITT) population includes all patients randomised onto the study.

## 6.2 Eligible study population

The eligible study population is all patients randomised onto the study excluding patients with gross eligibility deviations at either registration or randomisation. Patients with gross eligibility deviations will be determined in consultation with the Chief Investigator.

## 6.3 Per protocol population

The per protocol (PP) population includes all eligible patients who, after randomisation, take trial treatment adequately as per protocol (which will be determined retrospectively in a blinded fashion).

### 6.4 Run-in stage population

The run-in stage population includes all patients registered onto the study, i.e. all those consenting to participate in the run-in stage.

### 6.5 Safety population

The safety population includes all patients who, after randomisation, start trial treatment (whether they receive ketamine or placebo).

## 7 TABULATIONS, FIGURES AND LISTINGS

Throughout this section the tabulations consist of counts and percentages per category unless otherwise stated. Listings will include the patient identifier, study arm and the specific data for that listing.

### 7.1 Study Recruitment

a) A plot of actual cumulative recruitment against time since study opened will be provided. The target recruitment rate line will be superimposed on this plot.
b) A table of recruitment by centre will also be provided showing the date the centre opened, the date the first and last patients were recruited and the number recruited. Centres that have opened but not recruited patients will also be incorporated in this table.

### 7.2 Form Return

The following tables are only provided as part of interim analysis reports.

a) A table showing the number of forms due, the number of forms outstanding and the percentage of forms overdue by form type and study arm.
b) A table showing the number of forms due, the number of forms outstanding and the percentage of forms overdue by centre.

### 7.3 Patient progress

A CONSORT[1] style flow diagram showing the progress of patients through the phases of the study (enrolment, run-in, intervention allocation, titration, assessment, run-out and data analysis) will be presented.

### 7.4 Eligibility/ population membership

As this trial involves a pre-randomisation run-in period, there are eligibility criteria for both registration and randomisation. These will be considered separately below.

#### 7.4.1 Registration eligibility

All the data presentations in this section are based on the run-in population.

a) The factors considered for the inclusion and exclusion criteria on the registration form will be tabulated.
b) The waiver details of patients given a waiver to be registered onto the study will be listed, including details of whether or not the patient was subsequently randomised onto the trial.

#### 7.4.2 Randomisation eligibility

a) For patients who were registered but not randomised onto the study, the reason for not being randomised will be listed.

b) The factors considered for the inclusion and exclusion criteria on the randomisation form will be tabulated.
c) The waiver details of patients given a waiver to be randomised onto the trial will be listed.

#### 7.4.3 Population membership

a) Membership of the eligibility population ("eligible"/"ineligible") will be tabulated by treatment arm.
b) Ineligible patients will be listed together with the reasons for ineligibility.
c) Membership of the per protocol population ("included"/ "excluded") will be tabulated by treatment arm.
d) Membership of the safety population ("included"/ "excluded") will be tabulated by treatment arm.
e) Patients excluded from the safety population will be listed together with the reason for not starting trial treatment and whether or not the patient was eligible.

### 7.5 Baseline data and demographic characteristics

#### 7.5.1 Trial accrual

Patient accrual by centre [stratification factor] and whether the patient was randomised or did not participate beyond registration will be tabulated. A graph of the cumulative number of patients randomised against the target number will also be presented.

#### 7.5.2 Run-in period

As a summary of the run-in period of the trial, the following will be tabulated:

a) Worst Index Site Pain Score (VAS) at start and end of run-in period (mean, standard deviation, median inter-quartile (iq) range and range).
b) Number of days in run-in (mean, standard deviation, median inter-quartile (iq) range and range).
c) Details (name, route, prescribed dose, unit, schedule, start date and stop date (or "continuing") of adjuvant analgesic use throughout the run-in period will be listed.
d) Future study participation will be tabulated. For patients not being considered for randomisation, a listing of the reasons will be produced along with a listing of specifications for "other" reasons.
e) Summary of end of run-in pain score data from EuroQoL, Distress Thermometer, HADS, SF-MPQ, LANSS (mean, standard deviation, median inter-quartile (iq) range and range).
f) The number of patients completing the end of run-in period HADS, SF-MPQ and LANSS will be tabulated. Reasons for not completing these will be listed along with specifications for "other" reasons.

#### 7.5.3 Baseline data at randomisation

As a summary of patient characteristics at randomisation, the following details will be tabulated by treatment arm:

a) Age at registration [stratification factor]
b) Gender [stratification factor]
c) Baseline McGill sensory pain score [stratification factor]
d) Type of previous analgesic [stratification factor]

The mean, standard deviation, median inter-quartile (iq) range and range will be calculated for a) and c).

As a summary of patient characteristics before starting allocated intervention, the following pre-treatment data will be tabulated by treatment arm:

a) Weight (mean, standard deviation, median inter-quartile (iq) range and range).

b) Ethnicity, with specification for “other”.
c) The site of the primary tumour, with specification for “other”.
d) Presence of metastases, with the site and specification for “other” if applicable.
e) Cancer history: Prior chemotherapy, prior radiotherapy and prior hormonal therapy, with listing of details.
f) History of painful neuropathy and history of chronic pain will be tabulated: No; Yes, not problematic in the last 3 months; Yes, problematic in the last 3 months. For patients where these have been problematic in the last 3 months details will be listed.
g) Whether or not the patient has a history of drug or alcohol abuse. For patients with history further details will be listed.
h) Whether or the patient is currently alcohol or drug dependent.
i) Details of current medical conditions will be listed.
j) Baseline pain data from the SF-MPQ, VAS, LANSS (mean, standard deviation, median inter-quartile (iq) range and range).
k) Site, primary method of diagnosis, and association with metastatic site.
l) Worst score in the past 24 hrs for index neuropathic pain will be summarised (mean, standard deviation, median inter-quartile (iq) range and range).
m) All questions on the BTPQ will be tabulated (frequencies, mean, standard deviation, median inter-quartile (iq) range, range – as appropriate).

## 7.6 Delivery of study therapy

### 7.6.1 Titration period

As a summary of the delivery of study therapy in the titration period of the trial, the following will be tabulated by study arm:

a) Number of days in titration will be summarised (mean, standard deviation, median inter-quartile (iq) range and range).
b) Dose level at end of titration.
c) The number of days for which a patient took less than 4 doses of study medication, with listing of reason.
d) The number of days for which a patient took more than 4 doses of study medication, with listing of reason.
e) The number of times a dose reduction took place for each patient, with listing of reason.
f) Whether or not the patient is continuing to the assessment period will be tabulated: Yes; No, patient request; No, investigator decision; No, toxicity; No, other. Further specification for the “No, other” category will be listed.

### 7.6.2 Assessment period

As a summary of the delivery of study therapy in the assessment period of the trial, the following will be tabulated by study arm:

a) Number of days in assessment will be summarised (mean, standard deviation, median inter-quartile (iq) range and range).
b) A cross tabulation of dose level at beginning of assessment and dose level at end of assessment.
c) The number of days for which a patient took less than 4 doses of study medication, with listing of reason.
d) The number of days for which a patient took more than 4 doses of study medication, with listing of reason.

e) The number of times a dose reduction took place for each patient, with listing of reason.

f) Whether or not the patient completed the 16 day assessment period.

g) Whether or not the patient is continuing to the run-out period will be tabulated: Yes; No, patient request; No, investigator decision; No, toxicity; No, other. Further specification for the "No, other" category will be listed.

#### 7.6.3 Run-out period

As a summary of the delivery of study therapy in the run-out period of the trial, the following will be tabulated by study arm:

a) Number of days in run-out will be summarised (mean, standard deviation, median inter-quartile (iq) range and range).

b) The number of days for each patient where the actual dose taken was less than protocol dose, with listing of reason.

c) Whether or not the patient completed the run-out period.

### 7.7 <u>Opioid Use</u>

a) Number of patients taking any opioid medication will be tabulated by study period (run-in, titration, assessment) and study arm (Arm A, Arm B, Not allocated).

b) Details (name, route, prescribed dose, unit, schedule, start date and stop date (or "continuing") of opioid use will be listed by study arm and study period.

c) The normal number of breakthrough doses of opioid medication per day in the run-in period will be tabulated (mean, standard deviation, median inter-quartile (iq) range and range).

d) The proportion of days during the run-in period that a patient required breakthrough opioids will be summarised (mean, standard deviation, median inter-quartile (iq) range and range).

e) The proportion of days during the run-in period that a patient required more than 2 doses above normal breakthrough opioids will be summarised (mean, standard deviation, median inter-quartile (iq) range and range).

### 7.8 <u>Unblinding</u>

The following will be tabulated by study arm:

a) Number of patients unblinded, with listing of reason.

b) System used to perform unblinding.

c) Number of cases of unblinding discussed with Chief Investigator, with listing of reasons where there was no discussion.

### 7.9 <u>Safety analyses</u>

#### 7.9.1 Run-in period

Based on the run-in stage population: Opioid toxicity will be tabulated, with a listing of details (short name, worst grade observed, outcome and whether or not the event was a SAE) and a specification of "other" toxicities.

#### 7.9.2 Adverse events

The data presentations in this section relate to the titration, assessment and run-out periods and are based on the safety analysis population. All adverse events will be graded according to the NCI CTCAE v3.

a) The worst grade of each listed AE will be tabulated by study arm.
b) The worst grade of "other" toxicities that graded ≥ grade 2 by at least 5% of patients will be tabulated.
c) The details (Patient identifier, study arm, short name for AE, CTC grade, relationship to study drug and whether or not a SAE) for all adverse events graded as 3/4 will be listed.

#### 7.9.3 Serious adverse events/reactions/SUSARs

Details of serious adverse/events/reactions and SUSARs will be provided in a separate document output from the CTU’s pharmacovigilance database.

### 7.10 Efficacy analyses

Primary and secondary efficacy analyses will be conducted on the ITT populations.

Box-and-whisker plots will be produced of the following variables by study time-point (by day, e.g. Day1 Titration, Day2 Titration, …….., Day1 Assessment, …. etc) and study arm:

SF-MPQ, LANSS, HADS, EuroQol, Distress Thermometer, global pain score and index neuropathic pain.

Box-and-whisker plots of change from baseline by study arm will also be produced for all of the variables listed above.

The number of patients at each time-point will be indicated on all box-and-whisker plots.

#### 7.10.1 Primary efficacy analysis

The estimated difference in the “success” rate between the study arms at the day 16 assessment point will be tabulated along with the associated 95% confidence interval. A Kaplan-Meier plot of time to treatment failure, with ‘Proportion of successes’ as the y-axis, will be given. The p-value associated with the log-rank comparison of the survival curves will be given on this plot.

This analysis will be performed twice:

- Primary analysis: Where a significant change in opioid dose based only on the prescribed background opioid dose is used in the definition of a treatment “failure”.
- Exploratory analysis: Where a significant change in opioid dose based on the prescribed background opioid dose plus recorded breakthrough (PRN) dose is used in the definition of a treatment “failure”.

A secondary confirmatory analysis of the primary analysis will be based on a Cox regression model incorporating the study stratification factors as covariates. The p-value, hazard ratio and associated 95% confidence interval for study arm will be tabulated.

As part of the assessment of the primary end-point the number of patients with a significant change in opioid dose (>30% increase in 24 hour morphine equivalent daily dose during titration or assessment period) during the titration or assessment period, defining them as a treatment “failure”, will be tabulated. This data will be reviewed and confirmed by the CI blinded to treatment arm.

#### 7.10.2 Secondary efficacy analysis

a) The primary efficacy analysis will be repeated on the PP population.
b) The differences in the initial treatment benefit rates (using the “success” rate at the day 4 assessment point) between the study arms will be estimated and tabulated along with the associated 95% confidence interval. The p-value for the comparison will also be given.
c) The number of patients completing the pain score questionnaires (EuroQol, Distress, HADS, SF-MPQ, and LANSS) at the end of the titration period and days 4, 8, 12 and 16 of the assessment period will be tabulated. A listing of reasons for not completing the questionnaires will be given.

d) The adjusted standardised AUC (AUC calculated over the assessment/titration period, divided by number of days on assessment/titration and with the baseline value subtracted) of the following efficacy measures will be tabulated (mean, standard deviation, median inter-quartile (iq) range and range) by study arm. The difference in the median AUC will be given together with the associated 95% confidence interval and estimated p-value. All of the above will be produced for
   - Global pain
   - Neuropathic pain (LANSS pain scale)
   - Index neuropathic pain (worst pain score base on VAS)
   - patient distress (based on NCCN Distress Thermometer)
   - quality of life scores (based on Euroqol thermometer)
   - anxiety and depression (based on HAD scale)
e) In consultation with the CI the average daily opioid requirement over the titration and assessment period for the patients will be calculated in terms of morphine equivalent daily dose. This will be summarised by treatment arm (mean, standard deviation, median inter-quartile (iq) range and range). The estimated difference in the medians will be given together with the associated p-value and 95% confidence interval.
f) Side-effects and tolerability of trial drug – covered by §7.6.
g) The method for summarising the effect of intervention on sensory changes (as assessed by Quantitative Sensory Testing (QST)) will be defined after discussion with the Chief Investigator.
h) All questions on the BTPQ will be tabulated as a comparison of the proportion of patients improving between the arms.

### 7.11 Concomitant medication
Details of concomitant medication taken by patients will be listed.

## 8 STATISTICAL METHODS FOR THE ANALYSIS OF THE STUDY DATA

### 8.1 Primary efficacy analysis
The primary comparison will be in terms of time to treatment "failure" (as defined in §4.1) between the study arms. This comparison will be made using the log-rank test. A 95% confidence interval for the difference in proportions at day 16 will be derived using "Method 10" as described in R.G. Newcombe[(1)].

### 8.2 Secondary efficacy analyses
Initial treatment benefit, using the sensory component of the SF-MPQ, will be assessed by comparing the "success rates" at day 4 of assessment period between the treatment arms using the chi-square test. A 95% confidence interval for the difference in rates at day 4 will be derived using "Method 10" as described in R.G. Newcombe[(2)].

The analysis of VAS pain score will be complicated by missing data. Analysis of this data will use AUC techniques[(3)]. Missing values will be filled in using interpolation or last value carried forward as appropriate. (Note that this is different from the multiple imputation approach stated in the protocol - the different assessment and titration period durations for the patients make multiple imputation impractical).

The adjusted standardised AUC will be compared between the arms using the Mann Whitney U-test. Bootstrap methods will be used to estimate the median difference and associated 95% confidence interval.

The analysis of HADS, Quality of Life (EuroQol thermometer), LANSS and the NCCN Distress Thermometer, as for VAS above.

The average morphine use will be compared between the arms using the Mann-Whitney U-test. Bootstrap methods will be used to estimate the median difference and associated 95% confidence interval.

The worst grade of tabulated AEs will be compared between the arms using the Mann-Whitney U test.

The comparison of the proportions improving on the BTPQ will be made using the chi-square test.

### 8.3 Interim analyses

The study data will be reviewed annually by a DMEC, primarily from a safety standpoint. There will be one formal interim analysis for efficacy after half the patients have been assessed. A p-value <0.001 in the treatment comparison will trigger consideration of study closure or modification by the DMEC.

## 9 REFERENCES


1. Schulz KF, Altman DG, Moher D, for the CONSORT Group. CONSORT 2010 Statement: updated guidelines for reporting parallel group randomised trials. Trials 2010, 11:32. http://www.trialsjournal.com/content/11/1/32 (accessed 13 March 2011)
2. R.G. Newcombe. Interval Estimation for the Difference Between Independent Proportions: Comparison of Eleven Methods. Statistics in Medicine 1998; 17 (8): 873-90.
3. Qian W, Parmar MKB, Sambrook RJ, Fayers PM, Girling DJ, Stephens RJ. Analysis of messy longitudinal data from a randomised clinical trial. Statistics in Medicine. 2000; 19: 2657-74.
